# Burden of paediatric influenza in Western Europe: a systematic review

**DOI:** 10.1186/1471-2458-12-968

**Published:** 2012-11-12

**Authors:** Evgeniya N Antonova, Catherine E Rycroft, Christopher S Ambrose, Terho Heikkinen, Nicola Principi

**Affiliations:** 1 MedImmune, LLC, 1 MedImmune Way, Gaithersburg, Maryland 20878, USA; 2 RTI Health Solutions, 2nd Floor, The Pavilion, Towers Business Park, Wilmslow Road Didsbury, Manchester, M20 2LS, United Kingdom; 3 Department of Paediatrics, University of Turku and Turku University Hospital, FI-20520, Turku, Finland; 4 Department of Physiopathology and Transplantation, University of Milan, Fondazione IRCCS Ca' Granda, Ospedale Maggiore Policlinico, I-20122, Milan, Italy

**Keywords:** Influenza, Children, Vaccination, Europe, Burden

## Abstract

**Background:**

Influenza illness in children causes significant clinical and economic burden. Although some European countries have adopted influenza immunisation policies for healthy children, the debate about paediatric influenza vaccination in most countries of the European Union is ongoing. Our aim was to summarise influenza burden (in terms of health outcomes and economic burden) in children in Western Europe via a systematic literature review.

**Methods:**

We conducted a systematic literature search of PubMed, EMBASE, and the Cochrane Library (1970-April 2011) and extracted data on influenza burden in children (defined as aged ≤ 18 years) from 50 publications (13 reporting laboratory-confirmed influenza; 37 reporting influenza-like illness).

**Results:**

Children with laboratory-confirmed influenza experienced hospitalisations (0.3%-20%), medical visits (1.7-2.8 visits per case), antibiotic prescriptions (7%-55%), and antipyretic or other medications for symptomatic relief (76%-99%); young children and those with severe illness had the highest rates of health care use. Influenza in children also led to absenteeism from day care, school, or work for the children, their siblings, and their parents. Average (mean or median) length of absence from school or day care associated with confirmed influenza ranged from 2.8 to 12.0 days for the children, from 1.3 to 6.0 days for their siblings, and from 1.3 to 6.3 days for their parents. Influenza negatively affected health-related quality of life in children with asthma, including symptoms and activities; this negative effect was smaller in vaccinated children than in non-vaccinated children.

**Conclusions:**

Influenza burden in children is substantial and has a significant direct impact on the ill children and an indirect impact on their siblings and parents. The identified evidence regarding the burden of influenza may help inform both influenza antiviral use in children and paediatric immunisation policies in European countries.

## Background

Influenza is a highly contagious infectious disease that is responsible for between 3 and 5 million cases of severe influenza illness each year [1]. Many countries’ immunisation guidelines recommend influenza vaccination of the elderly and individuals with underlying chronic medical conditions because of their increased risk of complications due to influenza [2]. However, it is often forgotten that children have the highest influenza attack rates, with annual incidence rates of up to 30% [3]. This translates into significant illness and health care resource use, particularly related to outpatient consultations and hospitalisations [4-8]. Consequently, paediatric influenza leads to substantial economic and societal burdens [4,7,9-11].

Children not only incur significant morbidity associated with influenza but also are primary vectors of influenza transmission in the community [12-15] because they have limited pre-existing immunity, shed virus at higher viral titers and for a longer period than adults [16], are in close contact with one another in schools and other settings, and can have poor hygiene habits [17]. Dynamic transmission modelling (a tool that can compute the effect of infectious disease transmission) and several community-based studies have demonstrated that paediatric influenza vaccination, in addition to direct benefit to the vaccinated children, can also indirectly protect other members of the community [15,18-23].

Multiple countries recognise the burden of paediatric influenza and the role of children in disease transmission and recommend paediatric vaccination. The United States Advisory Committee on Immunization Practices has recommended annual vaccination of all children aged 6 months and older since 2008 [9]. Canada, Hong Kong, several Latin American countries, China, Singapore, Korea, and Taiwan also recommend paediatric vaccination, although most policies in these countries are restricted to young children (i.e., those aged ≤ 2 years or aged ≤ 5 years, depending on the country) [24-31]. Finland recommends vaccination of all children aged 6 months to 35 months and has implemented a fully-funded childhood immunisation programme since 2007. Latvia and Slovenia recommend vaccination of children aged 6 months to 2 years. Austria, Estonia, Slovakia, and Saxony in Germany recommend vaccination of children aged 6 months to 18 years [32,33]. In the United Kingdom, the Joint Commission on Vaccination and Immunisation has recently recommended that the influenza vaccination program should be extended to low risk children aged 2 to less than 17 years [34].

Although influenza burden has been studied in many countries, including some European countries [35-38], the overall burden of seasonal influenza in multiple countries in Western Europe has not been summarised in a systematic manner. Furthermore, although ample data on influenza burden are available for adults – particularly the elderly and individuals with comorbidities – such data for children are sparse. A systematic summary of influenza burden in Western Europe could help inform the paediatric immunisation policy debate [2]. Our aim was to summarise influenza burden (in terms of health outcomes and economic burden) in children in Western Europe via a systematic literature review.

## Methods

To retain a manageable volume of literature, this study included eight Western European countries: Austria, Finland, France, Germany, Italy, the Netherlands, Spain, and the United Kingdom (UK). We selected countries known for their influence on decision-making in the European Union (EU), paediatric immunisation policies, or previous epidemiologic investigations of paediatric influenza. We conducted a systematic and comprehensive search of medical literature electronically indexed in PubMed, EMBASE, and the Cochrane Library. We used a detailed search strategy and combined free-text search terms with Medical Subject Headings. The search terms were related to influenza, cost or burden analyses, health-related quality of life (HRQoL), absenteeism, and productivity (see Additional file 1: Table S1 in the Online Appendix for more details). Our search methodology complied with Preferred Reporting Items for Systematic Reviews and Meta-analyses (PRISMA) requirements [39].

Our search included studies conducted in children (defined as aged ≤ 18 years) and published since January 1970. We retained only studies that reported data on costs, resource use, absenteeism, and/or impact on HRQoL associated with influenza specifically for children; however, data on absenteeism covered both enrolled children and their parents in some studies. We excluded publications related to the H1N1 pandemic in 2009-2010 because pandemic influenza is substantially different from seasonal influenza. We also excluded publications relating to influenza-like illness (ILI) and retained only those publications reporting laboratory-confirmed influenza, as the latter assessment method is more specific than all-cause ILI. For the purposes of this article, we refer to “seasonal influenza” as “influenza”. We limited publications by excluding any editorials, letters, meta-analyses, practice guidelines, and comments. We examined references of included articles to find additional relevant publications for the review. We also searched Web sites of the health technology assessment bodies of the eight countries of interest for relevant influenza vaccine appraisals.

**Figure 1:**
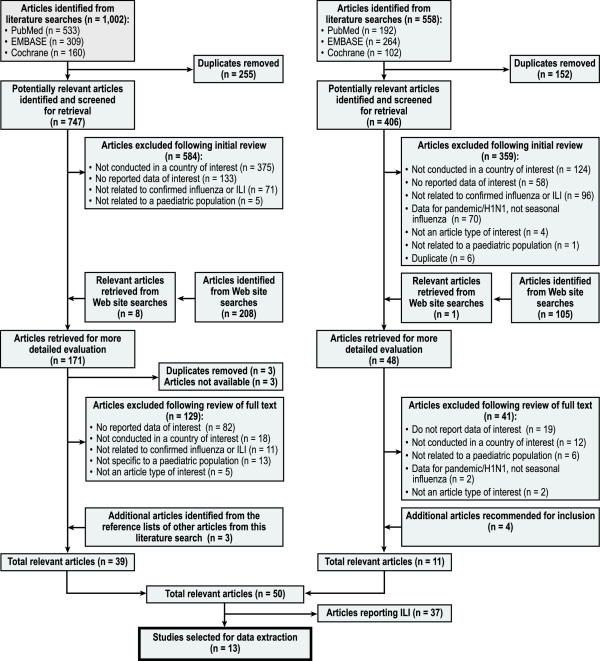
**PRISMA flow diagram of literature review for two searches: March 2009 review and April 2011 update.** ILI = influenza-like illness; PRISMA = Preferred Reporting Items for Systematic Reviews and Meta-analyses.

Two independent reviewers (CR and one other researcher at RTI Health Solutions) screened the identified publications according to pre-defined inclusion and exclusion criteria described above. We next conducted our review in two phases. In Phase 1, the reviewers screened all the titles and abstracts and selected publications for a full-text review. In Phase 2, the reviewers examined the full text of the selected articles and determined the articles’ concordance with the study’s research aims. To control quality, both reviewers compared their results and resolved all discrepancies at each phase. We fully documented the inclusion and exclusion processes using a PRISMA flow chart that detailed the number of included and excluded articles [40] (Figure 1).

We summarised data in evidence tables by the following domains: (1) health care resource use, (2) costs (including direct medical costs, direct non-medical costs, and indirect [societal] costs), (3) HRQoL, and (4) absenteeism and loss of productivity in patients and their families. We excluded studies not specific to children. We also extracted data on the incidence of influenza and of complications of influenza (e.g., acute otitis media, pneumonia, and lower respiratory tract infections) as secondary outcomes from the articles on economic burden. We recorded the following influenza assessment methods: via laboratory methods (virology or polymerase chain reaction) or via a list of ILI symptoms. However, we report only studies of laboratory-confirmed influenza, and the ILI studies are summarised in the supplementary appendix (see Additional file 1: Table S2 in the Online Appendix).

We assessed the quality of published studies according to the Oxford Centre for Evidence-based Medicine Levels of Evidence [41]. Finally, we assessed each study’s risk of bias, particularly focusing on the study population (community members or a particular subset of patients). An independent quality-control specialist confirmed data-extraction accuracy.

## Results

The results below represent studies of laboratory-confirmed influenza. Results from studies of ILI can be found in Additional file 1: Table S2 in the Online Appendix.

### Articles included

Figure 1 presents the details of the literature search and review process. From the initial literature search (conducted in 2009), we identified 747 unique, potentially relevant articles. After Phase 1 screening of titles and abstracts and searches of relevant Web sites, 171 articles were considered for more detailed evaluation. Of these, three articles were duplicates, three articles could not be sourced, and a further 129 articles were excluded at Phase 2 screening. Another three articles were identified in the reference lists of the included articles. Therefore, a total of 39 articles were eligible for inclusion.

From the literature search update (conducted in 2011), we identified a total of 406 unique, potentially relevant articles. After Phase 1 screening and searches of relevant Web sites, 48 articles were considered for detailed evaluation, and of these, we excluded 41 articles at Phase 2 screening. Another four articles were identified from prior knowledge of one of the authors; thus, 11 additional articles were eligible for inclusion. In total, 50 articles were eligible for inclusion from the two stages of the review. Of those, 13 articles reported relevant data for children with laboratory-confirmed influenza, and the remaining 37 articles reported data on ILI.

Table 1 describes the study details, quality, and the risk of bias for the 13 articles that included data based on laboratory-confirmed influenza in children. Most studies investigated the burden of confirmed influenza in children 15 years of age or younger, and children’s ages varied among studies.

**Table 1:** Reviewed articles on culture-confirmed influenza in children

**Reference**	**Study design**	**Study settings Children age**	**Sample**	**Study quality**^**a**^**Study limitations/bias**	**Type of data**
**Finland**					
Heikkinen et al., 2004 [35]	Prospective, observational study of respiratory infections in community-based children.	Community (day care centres, family day care, and schools);	2,231 child-seasons,	1b: prospective cohort study;	Resource use, absenteeism
			382 episodes of culture-confirmed influenza were documented		
				Only winter months were evaluated.	
	Study seasons: 9 October 2000 — 20 May 2001 and 1 October 2001 — 19 May 2002.	≤ 13 years.			
	Follow-up: not specified.				
Heinonen et al., 2010 [42]	Randomised, double-blind, controlled trial comparing oseltamivir with placebo for clinical efficacy in children with influenza.	Community;	1,185 children were recruited in the community prior to influenza seasons; among those,409 children with fever or respiratory infection who attended the study clinic were randomised to either intervention or placebo; among those,98 (24.7%) children had laboratory-confirmed influenza	1b: randomised, controlled trial;	Resource use, absenteeism.
		1-3 years			
				Not a population- based study;^b^	
				Broad exclusion criteria prior to enrolment in the trial.	
	Study seasons: 2 local influenza circulation seasons (14 January — 9 April 2008 and 7 January — 26 March 2009).				
	Follow-up: 21 days.				
**France**					
Ploin et al., 2003 [43]	Prospective, observational study in a paediatric ED of a university hospital.	Paediatric ED;	304 infants consecutively enrolled during influenza peak	2b: prospective cohort study with poor follow-up;	Resource use, absenteeism
		≤ 11 months.			
			99 (33%) with confirmed influenza.	Not a population- based study.	
	Study season: 4 weeks of local influenza epidemic peak (weeks 3-6 in 2002).				
	Follow-up: 15 days.				
Ploin et al., 2007 [36]	Prospective, observational study in a paediatric ED of a university hospital.	Paediatric ED;	575 children consecutively enrolled during influenza peak	2b: prospective cohort study with poor follow-up;	Resource use, absenteeism
		< 36 months.			
			283 (49%) with confirmed influenza.	Not a population- based study.	
	Study season: 4 weeks of local influenza epidemic peak (weeks 3-6 in 2002).				
	Follow-up: 15 days.				
Sanni et al., 2004 [44]	Prospective, observational survey of hospitalised children.	Hospital;	114 nasal swabs collected; among those – 59 (51.8%) with confirmed influenza.	1b: prospective cohort study;	Resource use.
		≤ 15 years.			
				Not a population- based study.	
	Study season: 37 days of local influenza epidemic (1 January — 6 February 2002).				
	Follow-up: not specified.				
**Germany**					
Ehlken et al., 2005 [45]	Cost-of-illness analysis of a prospective, multi-centre, population-based epidemiological study on the impact of LRTI in children.	Office-based PCP and hospitals;	3,458 cases with LRTI, including 1,329 office based cases, 2,039 hospitalized cases, and 90 nosocomial cases.	2c: outcomes research;	Cost (direct and indirect).^c^
				Not a population- based study;	
		≤ 36 months.			
				Limited to children with LRTI;	
				Costs were imputed based on existing standards.	
	Study period: 2 years (1 November 1999 — 31 October 2001).				
	Follow-up: not specified.				
**Italy**					
Bosis et al., 2005 [46]	Prospective, observational, single-centre study of children enrolled at an ED, comparing the impact of confirmed influenza and RSV with hMPV.	ED;	All children (n = 1,505) attending the ED on Wednesdays and Sundays.	1b: prospective cohort study;	Resource use, absenteeism.
		< 15 years.			
				Not a population- based study.	
			Of these, 1,019 children had evidence of acute respiratory infection.		
			Influenza was confirmed by PCR in 230 (15.3%) of total cases; among these, 7 cases were co-infected with RSV or hMPV.		
	Study season: 5 months (1 November 2002 — 31 March 2003).				
	Follow-up: not specified.				
Esposito et al., 2005 [47]	Prospective, observational, single-centre study of children admitted to an ED, comparing the impact of confirmed influenza and RSV.	ED;	1,520 children attending ED for acute conditions other than trauma on Wednesdays and Sundays;	1b: prospective cohort study;	Resource use, absenteeism
		< 15 years.			
				Not a population- based study.	
			234 (15.4%) with confirmed influenza.		
	Study season: 5 months (1 November 2002 — 31 March 2003).				
	Follow-up: not specified.				
Esposito et al., 2011 [37]	Prospective, observational study of children presenting to PCP with ILI	PCP	PCPs continuously followed 21,986 community children	1b: prospective cohort study with good follow-up	Resource use, absenteeism, cost (direct and indirect)
		< 14 years			
			6,988 children with ILI presented to PCPs	Costs were imputed based on existing standards	
	Study season: 6 months (1 November 2008 —30 April 2009)		2,143 (30.7%) children had confirmed influenza		
	Follow-up: not specified				
Principi et al., 2003 [48]	Prospective, observational, multi-centre study.	ED and PCP;	3,771 children with ILI; among those	1b: prospective cohort study;	Resource use, absenteeism
		< 14 years.			
			352 (9.3%) with confirmed influenza, including 260 (8.7%) of 2,970 children seen in EDs and 92 (11.5%) of 801 children seen by PCPs	Not a population- based study.	
Principi et al., 2004 [38]	Study season: 6 months (1 November 2001 — 30 April 2002).				
	Follow-up: not specified.				
**The Netherlands**					
Bueving et al., 2004 [49]	Randomised, double-blind, placebo-controlled trial comparing inactivated vaccine with placebo for clinical efficacy in children with asthma.	Community;	696 children with asthma enrolled through PCP offices prior to influenza seasons’ start.	1b: individual randomised, controlled trial;	HRQoL.
		6-18 years.			
				Limited to children with asthma.	
	Study seasons: 2 influenza seasons (1999 — 2000 and 2000 — 2001).				
	Follow-up: not specified.				
Van Der Zalm, et al., 2009 [50]	Prospective birth cohort study, a part of a prospective, ongoing population-based birth cohort study on determinants of wheezing illness.	Community;	305 healthy full-term infants (2-3 weeks old);	2b: individual cohort study.	Resource use.
		≤ 1 year.			
			668 samples positively tested for any respiratory virus;		
			18 (2.7%) samples with influenza virus.		
	Study duration: October 2003 — September 2006.				
	Follow-up: until infants reached 1 year of age.				

ED = emergency department; hMPV human metapneumovirus; HRQoL = health-related quality of life; ILI = influenza-like illness; LRTI = lower respiratory tract infection; PCP = primary care paediatrician; PCR = polymerase chain reaction; RSV = respiratory syncytial virus.

^a^ Study quality according to the Oxford Centre for Evidence-based Medicine scale [41].

^b^ Studies that investigated the impact of influenza at the whole population level rather than the impact in a particular subset of patients (e.g., children admitted to hospital with fever).

^c^ Resource use, absenteeism, and HRQoL also reported but associated with ILI only (not associated with confirmed influenza).

Study quality and types varied. The 13 articles were classified as follows: two were randomised, controlled trials that had placebo groups (therefore providing data on influenza burden); seven were prospective cohort studies with good follow-up; three were prospective cohort studies with poor follow-up; and one was an ecological study.

### Clinical burden of influenza

#### Incidence of influenza

Studies reported influenza rates across a range of seasons and settings. Table 2 summarises influenza incidence and prevalence rates in various study settings. Annual incidence of influenza was 62 per 1,000 in full-term infants aged 1 year or younger [50]. Annual incidence of lower respiratory tract infection due to influenza was 1.1 per 100 children-years in children aged 36 months or younger [45]. During fall, winter, and spring, when infection risk is highest in European countries, influenza incidence rates were 167 per 1,000 in children aged 13 years or younger (9 October 2000 — 20 May 2001 and 1 October 2001 — 19 May 2002 in Finland [35]) and 96 per 1,000 children younger than 14 years (1 November 2008 — 30 April 2009 in Italy [37]).

**Table 2:** Incidence and prevalence rates of laboratory-confirmed influenza in children

**Reference**	**Country**	**Age**	**Setting**	**Study season or period**	**Influenza rates**
**Community-based estimates**					
Van der Zalm, et al., 2009 [50]	The Netherlands	< 1 year	Community	October 2003 – September 2006.	Prevalence rate: influenza was detected in 2.7% of respiratory samples
				Follow-up: until infants reached 1 year of age.	
					Annual incidence rate: 62 per 1,000^a^
Heikkinen et al., 2004 [35]	Finland	≤ 13 years	Community	9 October 2000 – 20 May 2001 and 1 October 2001 – 19 May 2002.	Influenza-season incidence rate per 1,000 children:
					All ages combined: 167,^b^
					Age < 3 years: 179,
					Age 3-6 years: 175,
					Age 7-13 years: 142.
**Health care setting-based estimates**					
Ploin et al., 2003 [43]	France	0-11 months	Paediatric ED	4 weeks of local influenza epidemic peak (weeks 3-6 in 2002)	Prevalence rate:
					Total: 33%,
					Aged 0-2 months: 31%,
					Aged 3-5 months: 27%,
					Aged 6-8 months: 30%,
					Aged 9-11 months: 40%.
Ploin et al., 2007 [36]	France	< 36 months	Paediatric ED	4 weeks of local influenza epidemic peak (weeks 3-6 in 2002)	Prevalence rate: 49%
Ehlken et al., 2005 [45]	Germany	0-36 months	PCP, paediatric hospital	1 November 1999 – 31 October 2001.	Annual incidence rate of LRTI associate with influenza: 1.1 per 100 children-years.
Heinonen et al., 2010 [42]	Finland	1-3 years (mean age: 2.4 years)	Primary care clinic	Two local influenza circulation seasons: 14 January – 9 April 2008 and 7 January – 26 March 2009.	Prevalence rate: 24.7% of children tested positive for influenza.
				Follow-up: 21 days.	
Esposito et al., 2011 [37]	Italy	< 14 years	PCP (with community “base”)	1 November 2008 – 30 April 2009.	Influenza-season incidence rate: 96.4 per 1,000 children.
Principi et al., 2003 [48]	Italy	< 14 years	PCP and ED	1 November 2001 – 30 April 2002.	Prevalence rate: 9.3%, (virology or PCR),
Principi et al., 2004 [38]					Including:
					8.7% of children seen in EDs and
					11.5% of children seen by PCPs.
Bosis et al., 2005 [46]	Italy	< 15 years	ED	1 November 2002 – 31 March 2003.	Prevalence rate; 15.3% (by PCR).
Esposito et al., 2005 [47]	Italy	< 15 years	ED	1 November 2002 – 31 March 2003.	Prevalence rate: 15.4% (by PCR).
Sanni et al., 2004 [44]	France	≤ 15 years	Hospital	37 days of local influenza epidemic (1 January – 6 February 2002)	Prevalence rate:
					Total: 51.8%,
					Aged 0-1 year: 42.4%,
					Aged > 1 and ≤ 3 years: 68.9%,
					Aged > 3 and ≤ 5 years: 41.2%,
					Aged > 5 and ≤15 years: 36.8%.

ED = emergency department; LRTI = lower respiratory tract infection; NR = not reported; PCP = primary care pediatrician; PCR = polymerase chain reaction

Note: For further details on study design and patient characteristics, see Table 1.

^a^ We calculated this rate from the paper as follows: 18 [influenza cases] ÷ (305 [enrolled children, corrected for dropout] × 11.5 [average months of follow-up per child] ÷ 12 [months in a year]) × 1,000 = 62.

^b^ We calculated this rate from the data reported in the paper (372 [influenza cases] ÷ 2,231 [recruited children] × 1,000 = 167).

Influenza was a common culture-confirmed pathogen in children seeking health care due to ILI. In a randomised, controlled trial of children with ILI, influenza was confirmed in 25% of children aged 1 through 3 years during an active epidemic period (14 January — 9 April 2008) in Finland [42]. In children presenting to the emergency department (ED) with ILI, influenza was identified in 33% (0-11 months old) and 49% (≤ 36 months old) during a 4-week local influenza peak (weeks 3 – 6 in 2002) in France [36,43]. In Italy, influenza was identified in children presenting to the ED during 5 winter months: 15% in those younger than 15 years (1 November 2002 — 31 March 2003 [46,47]) and in 8.7% of those younger than 14 years (1 November 2001 —31 March 2002 [38,48]). Among children hospitalised with ILI during 37 days of a local influenza epidemic 1 January — 6 February 2002), influenza was confirmed in 51.8% of those who tested negative for respiratory syncytial virus [44]. Among hospitalised children, the prevalence of influenza was highest in the toddler group (2-3 years; 68.9%), followed by infants (≤ 1 year; 42.4%), pre-school children (3-5 years; 41.2%), and school-aged children (6-15 years; 36.8%) [44].

#### Complications of influenza

**Figure 2:**
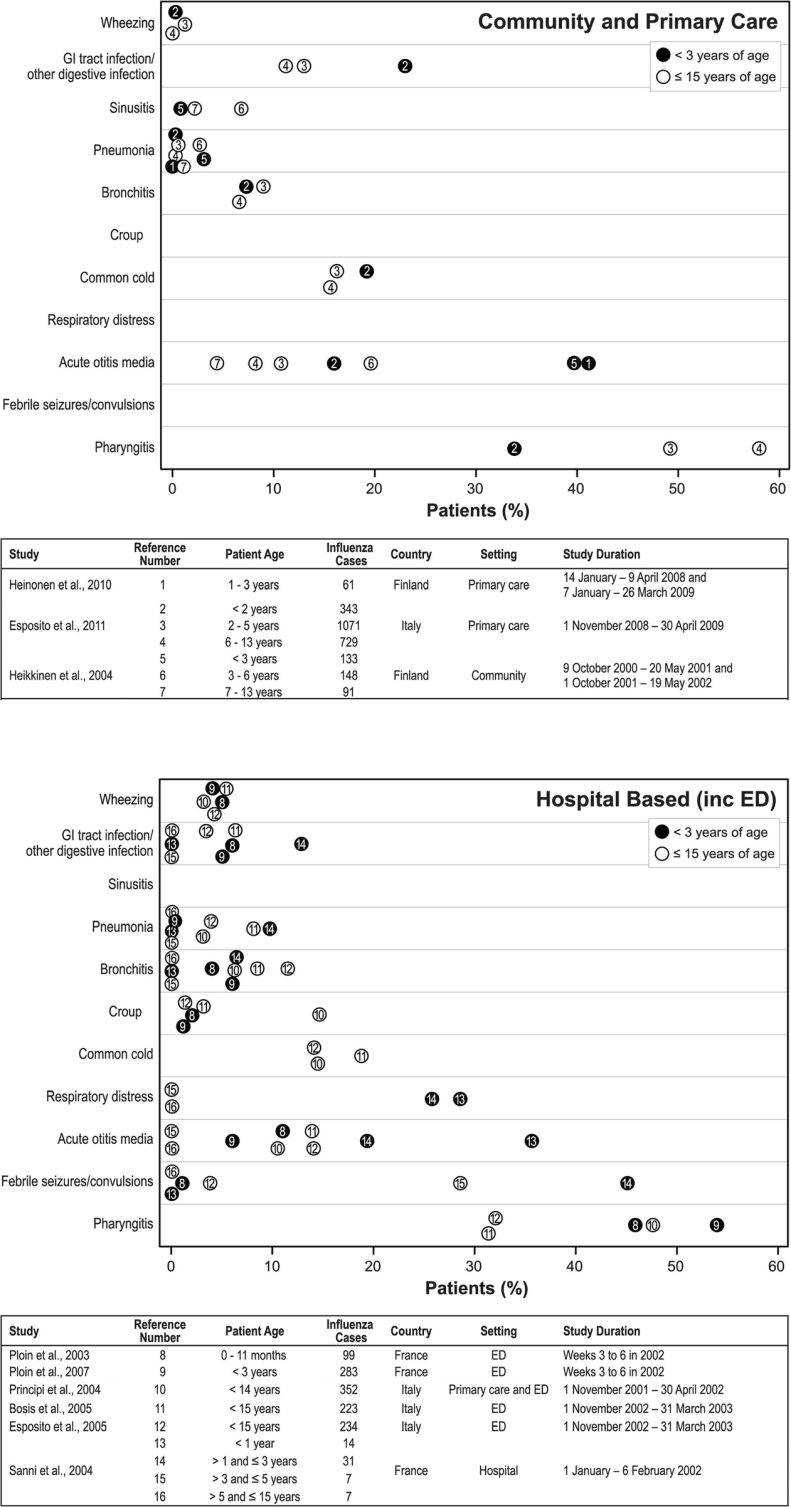
**Incidence of complications in children with laboratory-confirmed influenza.** ED = emergency department; GI = gastrointestinal. Notes: Confidence intervals were not presented for any of these values within any of the source articles. We calculated the rate of acute otitis media for the placebo group from the Heinonen et al. [42] article as follows: (6 [the number of patients with acute otitis media at baseline] + 19 [the number of the number of patients with new episodes of acute otitis media during the study]) ÷ 61 [the total number of patients in the placebo group] = 40.9%.

A wide range of complications occurred due to laboratory-confirmed paediatric influenza (Figure 2) [35-38,42-44,46,47,50]. Frequently reported complications included acute otitis media (10 articles), pneumonia (7 articles), bronchitis (7 articles), and wheezing (6 articles) (Figure 2). The most commonly occurring complications were pharyngitis (range: 31% - 58%), acute otitis media (range: 0% - 40.9%), and febrile seizures or convulsions (range: 0% - 45%) (Figure 2).

Febrile seizures [43,44,47], respiratory distress [44], and croup [36,43,46,47] were reported only in ED- or hospital-based studies. Wheezing and pneumonia were less prevalent in community- or primary care-based studies than in studies conducted in ED or hospitals (Figure 2). Respiratory distress was more common in children younger than 3 years than in those younger than 15 years [44].

### Economic burden

#### Health care resource use

**Figure 3:**
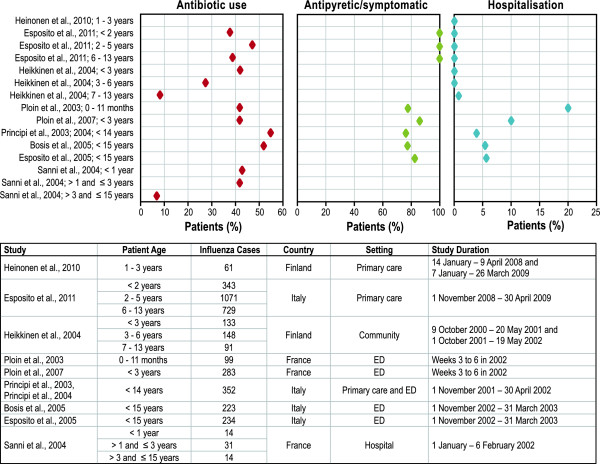
**Hospitalisations, antibiotic use, and antipyretic or symptomatic treatment use by children with culture-confirmed influenza.** ED = emergency department. Notes: Each point represents a percentage value reported in one of the identified studies. Confidence intervals were not presented for any of these values within any of the source articles.

**Figure 4:**
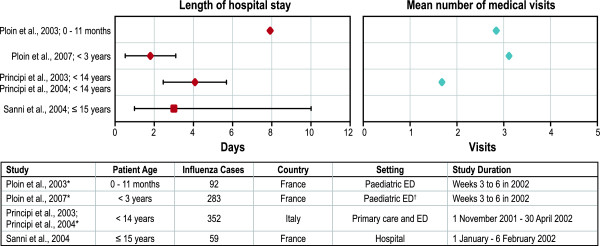
**Length of hospital stay and number of medical visits by children with culture-confirmed influenza.** ED = emergency department. * The mean number of medical visits = number of reported additional medical visits + initial 1 primary care or ED visit. ^†^ Length of stay in the ED, rather than in the hospital. Notes: Each point represents a mean or median value from one of the identified studies: ♦ = mean value; ▪ = median value. Effect sizes around each point represent standard deviation if value is a mean, range if value is a median.

Studies reported health care use primarily through hospitalisations, medical visits, and medications (antibiotic, antipyretic, or analgesic). Hospitalisation rates of children with laboratory-confirmed influenza ranged from 0% to 20% (Figure 3), and the mean length of stay ranged from 1.8 to 7.9 days (Figure 4). As would be expected, hospitalisation rates reported in physician office-based studies (0.7% [37]) and community studies (0.3% [35]) were lower than in ED-based studies (20% [43], 10% [36], 5.6% [47]) due to more severe influenza illness in children presenting to the ED than in those treated at a physician’s office. Two studies with identical country, influenza season, and health care settings demonstrated that infants (aged 0-11 months) presenting to the ED were more likely to be hospitalised (20%) and stayed in the hospital longer (mean: 7.9 days) [43] than children 0 through 3 years of age (10%; mean: 1.8 days) [36].

Infants and toddlers had a higher number of medical visits than children who were older (Figure 4). For example, in community-based studies, hospitalisation rates were 1.5% (for children < 2 years), 0.7% (2-5 years), and 0.4% (6-13 years) in Italy [37] and 0.8% (< 3 years), 0% (3-6 years), and 0% (7-13 years) in Finland [35]. A similar trend was observed in ED-based studies: children younger than 14 years had, on average, 1.7 medical visits [38]; by comparison, toddlers (< 3 years) had an average of 2.1 visits [36] and infants (< 1 year) had an average of 2.8 visits [43].

Medication use was high in children with influenza and varied by the study setting. Antibiotics were prescribed to 28% of children in community-based studies [35]; to 43% of children in primary care-based studies [37]; to 52% [50], 42% [36,43], or 55% [38] in the ED studies; and to 34% in hospital-based studies [44] (see Additional file 1: Table S3 in the Online Appendix). Furthermore, antibiotic use varied by age: infants and toddlers were more likely to receive antibiotics than older children. In a community-based study, the age trend of receipt of antibiotics was obvious: 42% (in children < 3 years), 28% (3-6 years), and 8% (7-13 years) [35]. The trend was similar, but less pronounced, in a primary care study (38% [< 2 years], 47% [2-5 years], and 38% [6-13 years] [37]) and in a hospital-based study (43% [< 1 year], 41% [1-3 years], and 7.1% [> 3years] [44]) (Figure 3). Most children with laboratory-confirmed influenza (76% - 99%) received antipyretics and other symptomatic treatment, with no obvious age-related trend (Figure 3).

Family members of children with influenza also consumed health care resources due to subsequent ILI (Additional file 1: Table S3 in the Online Appendix). For 10% to 43% of children with confirmed influenza, a similar illness was observed in a household member [37,38,46,47]. Between 0.3% and 0.4% of family members required hospitalisation [38,46-48], 5% to 8% received a prescription for antibiotics [38,46,47], 13% to 16% received antipyretics [38,46,47], and 10% to 14% required medical visits [46,47] (Additional file 1: Table S3 in the Online Appendix).

#### Costs

Multiple studies reported medical and societal costs associated with paediatric influenza, but only two reported results based on laboratory-confirmed illness (Table 3) [37,45]. In the first study, the annual total cost of paediatric community-acquired influenza in Germany was estimated at €7,530,105. This study considered children between 0 and 36 months of age with lower respiratory tract infection and estimated 2,913 influenza cases per year, with a median hospitalisation cost of €2,585 per case. Hospitalised paediatric influenza cases were more expensive than the office-based ones (€2, 597 vs. €223, year 2002 costs [45]).

**Table 3:** Costs associated with paediatric influenza

**Reference**, **country**, **and study period**	**Population**	**Costs**
Ehlken et al., 2005 [45],	3,458 children aged 0-36 months, with LRTI.	**Mean** (**SD**) **costs** (**in 2002 euros**) **per community**-**acquired**, **office**-**based case of confirmed influenza**:
Germany,	Setting: 11 office-based paediatricians and 5 hospitals.	Total cost: €223 (€280)
Influenza season: 1999 — 2001.		Direct medical cost: €66 (€24)
		Direct non-medical cost: €12 (€10)
		Indirect cost: €145 (€266)
		**Mean** (**SD**) **costs** (**in 2002 euros**) **per community**-**acquired**, **hospitalised case of confirmed influenza**:
		Total cost: €2,597 (€1,214)
		Direct medical cost: €2,428 (€1,200)
		Direct non-medical cost: €58 (€75)
		Indirect cost: €110 (€249)
		**Median** (**95**% **CI**) **annual economic burden** (**in 2002 euros**) **due to confirmed influenza**:
		Community-acquired office-based cases: NA
		Community-acquired, hospitalised cases:
		Median: €7,530,105 (€5,547,410-€10,011,705)
		Nosocomial cases: NA
Esposito et al., 2011 [37],	6,988 children aged < 14 years with ILI.	**Mean** (**SD**) **costs** (**in 2008 euros**) **of influenza in influenza**-**positive and influenza**-**negative children and their households**, **until resolution of illness**:
Italy,	Confirmed influenza cases: 2,143 (30.7%).	
Influenza season: November 2008 — April 2009.		Total: €131.70 (€71.40); €89.40 (€65.20); *P* < 0.001
	Setting: ED at a university hospital.	
		Paediatric examinations: €33.00 (€4.00); €30.60 (€4.20)
		Antibiotics: €3.70 (€4.30); €4.40 (€4.90)
		Antipyretics: €2.40 (€2.00); €1.90 (€1.40)
		Hospitalisation: €22.40 (€238.10); €22.50 (€251.00)
		Working days lost by mothers: €47.90 (€90.10); €26.70 (€89.90); *P* < 0.05
		Working days lost by fathers: €22.30 (€89.70); €3.30 (€39.90); *P* < 0.05
		**Mean** (**SD**) **costs of influenza in influenza**-**positive children with influenza A or influenza B and their households**, **until resolution of illness**:
		Total: €142.60 (€74.30); €72.80 (€53.30); *P* < 0.001
		Paediatric examinations: €33.30 (€4.60); €30.90 (€3.40)
		Antibiotics: €3.70 (€3.30); €3.40 (€3.10)
		Antipyretics: €2.50 (€2.10); €2.00 (€1.90)
		Hospitalisation: €22.40 (€243.40); €14.20 (€216.70)
		Working days lost by mothers: €54.40 (€94.80); €16.60 (€61.40); *P* < 0.05
		Working days lost by fathers: €26.30 (€97.70); €5.70 (€33.30); *P* < 0.05
		**Mean** (**SD**) **cost of influenza in influenza**-**positive children of different age groups (<2 years, 2-5 years, and 6-13 years) and their households**, **until resolution of illness:**
		Total: €153.20 (€72.80)^a^; €148.10 (€83.10)^a^; €73.90 (€41.90)
		Paediatric examinations: €33.50 (€5.60); €32.90 (€4.20); €33.00 (€2.50)
		Antibiotics: €3.20 (€3.90); €4.00 (€4.60); €3.30 (€3.90)
		Antipyretics: €2.40 (€1.90); €2.30 (€2.20); €2.10 (€2.50)
		Hospitalisation: €40.80 (€238.80); €23.90 (€268.90); €11.50 (€153.40)
		Working days lost by mothers: €46.70 (€96.40)^a^; €55.60 (€106.70)^a^; €19.80 (€49.60)
		Working days lost by fathers: €26.60 (€90.40)^a^; €29.40 (€111.40)^a^; €4.20 (€39.10)

CI = confidence interval; ED = emergency department; ILI = influenza-like illness; LRTI = lower respiratory tract infection; NA = not applicable; SD = standard deviation.

Note: For further details on study design and patient characteristics, see Table 1

^a^*P* < 0.05 vs. age > 5 years.

The second study was conducted in Italy and considered both direct (medical) and indirect (absenteeism) costs. The care for children with laboratory-confirmed influenza (averaging €132 per patient) was 32% more expensive than that for children with influenza-negative ILI [37]. An influenza hospitalisation cost approximately €3,000, and associated costs averaged €22.40 among all influenza cases (the total cost of hospitalization divided by the total number of children with influenza, regardless of their hospitalization status). The average indirect cost of working days lost by parents (€70) had the greatest impact on the average total cost of an influenza case. Furthermore, the cost of care for young children was greater than the cost of care for older children: €153 for children younger than 2 years, €148 for children aged 2 to 5 years, and €74 for children aged 5 to 13 years.

#### Absenteeism

**Figure 5:**
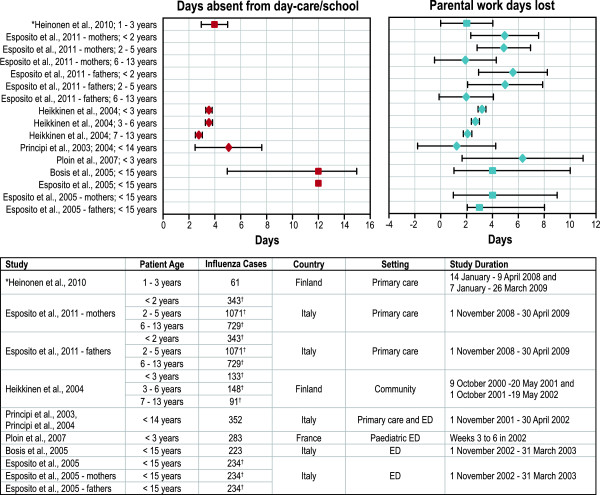
**Children’s absence from day care or school and parents’ absence from work associated with paediatric influenza.** ED = emergency department. * Interquartile range. ^†^ This is the number of children with confirmed influenza in this article, so the value is the same for the row showing the whole parent population, the mothers only, and the fathers only. The article does not provide the number of mothers and the number of fathers. Notes: Each point represents a mean or median value from one of the identified studies: ♦ = mean value; ▪ = median value. Effect sizes around each point represent standard deviation if the value is a mean; range represents minimum-maximum range if the value is a median.

Childhood influenza resulted in absenteeism for sick children, their siblings, and parents (Additional file 1: Table S3 in the Online Appendix and Figure 5). Absenteeism in ill children was assessed through absence from school or day care [35,38,42,46-48] or lost work or school days for the parents or siblings of infected children [35-38,42,43,46-48]. The rates of child absenteeism in the community-based study were 76% (in children < 3 years), 73% (3-6 years), and 77% (7-13 years) [35]; absenteeism was not assessed in other studies. The duration of absenteeism depended on influenza severity (Additional file 1: Table S3 in the Online Appendix). For example, children in ED-based studies were absent from school for a median of 12.0 days [46,47] and 5.1 days [38], children in physician office-based studies were absent from school for a median of 4.0 days [42], and children in community-based studies were absent from school for a mean of 3.6 days (calculated as weighted mean from Heikkinen et al. [35]). One study reported that some children missed up to 15 school days [46].

Parental absenteeism was common: between 11.2% and 61% of parents of children with laboratory-confirmed influenza took absence from work for their own influenza illness or to care for their children (Additional file 1: Table S3 in the Online Appendix). In Finland, parents were slightly more likely to take time off if their children were younger (61% [for children < 3 years], 54% [3-6 years], and 26% [7-13 years] [35]). However, the results were mixed in an Italian study (37.9% [for children < 2 years], 51.8% [2-5 years], and 32.5% [6-13 years] among mothers and 5.5% [for children < 2 years], 6.9% [2-5 years], and 2.5% [6-13 years] among fathers [37]) and two French studies (53% [for children 0-11 months] and 54% [for children < 36 months] [36,43]). The mean duration of work absenteeism for parents ranged from 1.3 to 6.3 days [35-38], and the median work absenteeism for parents ranged from 2 to 4 days [42,46,47].

Siblings of children with influenza also missed school days due to subsequent ILI. For example, siblings of children with influenza lost a mean of 1.3 days [38,48] or a median of 5.0 to 6.0 days [46,47] of school or work.

### Health-related quality of life

A single study reported HRQoL associated with confirmed influenza specifically in children [49]. That study (a prospective, randomised, double-blind, placebo-controlled trial) compared trivalent-inactivated vaccine with no vaccine. It assessed HRQoL in children with asthma through the Paediatric Asthma Quality of Life Questionnaire (range of possible scores: 1-7, with 7 indicating the highest HRQoL) [49]. Both the vaccinated and the placebo groups experienced worsening of asthma-related HRQoL. However, the worsening was less severe in children in the vaccinated group than in children in the placebo group, as measured by the total score (–0.40 vs. –1.0 points; *P* value = 0.02), the “activities” domain (–0.49 vs. –1.31 points; *P* value = 0.02), the “emotions” domain (–0.21 vs.–0.41 points; *P* value = 0.29), and the “symptoms” domain (–0.52 vs. –1.35 points; *P* value = 0.04). No other studies were identified that reported the quality-of-life impact of confirmed influenza specifically in children.

### Data availability by country

Data availability varied by country. Studies reporting burden of laboratory-confirmed influenza were conducted in Italy (5 articles), France (3 articles), Finland (2 articles), the Netherlands (2 articles), and Germany (1 article). No articles that reported burden data relating to confirmed influenza were identified from Austria, Spain, or the UK.

Data on health care resource use were most commonly reported in Italy and France, whereas costs of laboratory-confirmed influenza were reported for Germany and Italy. Absenteeism data were most commonly reported for Italy, whereas HRQoL impact of confirmed influenza was reported only in the Netherlands.

## Discussion

The current literature review provides a comprehensive summary of the available influenza burden data in children from selected Western European countries. The incidence and prevalence rates of ILI and laboratory-confirmed influenza, as well as the rates of complications summarised by our review, were similar to those reported in the United States and Asia [51-53]. The current analysis demonstrated that, along with substantial clinical burden, influenza results in a significant economic burden, including hospital stays, physician office visits, and use of antibiotics and antipyretics. Consistent with other literature, our study found that influenza also resulted in significant illness-related absenteeism among the enrolled children and their siblings and parents. These results concurred with a 2008 review of the literature from North America, Western Europe, Asia, and Australia: in studies from these countries, parental absenteeism ranged between 1.0 day and 5.9 days per influenza episode due to either a parent’s own illness or the child’s illness [54].

As expected, influenza burden depended on the severity of child’s illness (as reflected by the health care settings) and the child’s age. Children admitted to an ED or a hospital, in general, had more complications (wheezing, pneumonia, croup, respiratory distress, and febrile seizures or convulsions), were absent from school or day care for more days, and were more likely to receive an antibiotic prescription than their counterparts without these characteristics. Studies reporting health outcomes by children’s ages demonstrated that younger children were more likely to experience respiratory distress, child and parent absenteeism, hospitalisation, and antibiotic use than older children.

The small number of studies made cross-country comparisons difficult. It is challenging to draw fair cross-country comparisons between studies, because several other factors contributed to the studies’ variations in the results: age, health care setting, and influenza season. Differences in the paediatric health care systems across the EU also may contribute to difficulties in comparing and interpreting burden-of-disease information from different countries. For example, the UK, the Netherlands, and Finland have a general practitioner-led system; Spain has a paediatrician-led system; Italy, France, Germany, and Austria have a combination of the two in which school-age children can choose to see either a general practitioner or a paediatrician [55]. These differences in the health care systems may lead to differences in health care-seeking behaviours and ultimately to variance in influenza-reporting patterns [56].

The published literature had a number of limitations, primarily related to the design of the reported studies. Heterogeneity existed among influenza seasons, ages of studied children, and study methods for evaluating influenza illness. Additionally, all cost estimates were not directly collected but imputed (the rate of health care resource use multiplied by the reimbursement or standard prices associated with these resources) [37,45].

The findings of this review should be interpreted in the context of the limitations of the search criteria. We extracted data on complications and influenza incidence rates as secondary outcomes; we did not design a literature search to identify these data specifically. Furthermore, our study investigated eight selected countries rather than all EU countries.

The demonstrated burden of influenza in children underscores the need for a comprehensive approach to controlling its impact. Increased use of diagnostic tests for influenza would increase practitioners’ awareness of influenza and facilitate further epidemiologic research. Increased testing would also facilitate the use of influenza antiviral medications for treatment of influenza illness [42]. The demonstrated burden also highlights the potential benefits of the annual vaccination of children against influenza. Influenza immunisation policies in the EU vary. Although most countries recommend vaccinating elderly and high-risk individuals, only a minority of European countries recommend vaccinating the paediatric population (Austria, Estonia, Finland, Slovakia, Latvia, Slovenia, Saxony in Germany, and recently the UK) [31-34]. In light of the ongoing discussions about paediatric influenza vaccination by EU policy agencies [57], there is a need for a good understanding of the available evidence regarding the burden of influenza in European children.

## Conclusion

As indicated by several bodies, including the World Health Organization and the European Centre for Disease Prevention and Control, a better understanding of the burden of influenza is essential in informing influenza-related policy making [58-60]. Although there are certainly gaps in the existing data regarding the burden of influenza in children, the existing evidence demonstrates the significant burden influenza places each year on children and their families. Summaries of the available data may help facilitate decision-making in regard to both influenza antiviral use in children and childhood immunisation policies in EU countries [31,32].

## Competing interests

This study was sponsored by MedImmune, LLC, Gaithersburg, Maryland, USA. Dr Rycroft is a full-time employee of RTI Health Solutions. Dr Antonova and Dr Ambrose are full-time employees of MedImmune, LLC. Dr Principi has received funding through his institution to conduct studies for MedImmune, LLC, and has received advisory board fees from MedImmune, LLC. Dr Heikkinen has served as a consultant for MedImmune, LLC.

## Authors' contributions

CR led the literature searches, extracted the evidence from the source articles, and prepared the draft of the manuscript. EA contributed to the methodology and critical evaluation of the results and led writing, editing, and approval of the manuscript. TH, NP, and CA contributed to the critical interpretation of the results and provided feedback on the manuscript throughout its development. All authors read and approved the final manuscript.

## Pre-publication history

The pre-publication history for this paper can be accessed here:

http://www.biomedcentral.com/1471-2458/12/968/prepub

## Supplementary Material

Additional file 1 **Online appendix.Table S1.** Search Terms for PubMed, EMBASE, and the Cochrane Library Databases.** Table S2.** Studies reporting data not specific to culture-confirmed influenza. **Table S3.** Health care resource use and absenteeism in children with confirmed influenza. Click here for file
