# Supplementary material for: Burden of paediatric influenza in Western Europe: a systematic review
Source: BMC Public Health. 2012 Nov 12;12:968. doi: 10.1186/1471-2458-12-968 (PMC3534559; doi:10.1186/1471-2458-12-968)
Supplement: Additional file 1 — Online appendix.Table S1. Search Terms for PubMed, EMBASE, and the Cochrane Library Databases. Table S2. Studies reporting data not specific to culture-confirmed influenza. Table S3. Health care resource use and absenteeism in children with confirmed influenza. [file 1471-2458-12-968-S1.doc]

**Online appendix**

Supplemental File 1 - Search Terms for PubMed, EMBASE, and the Cochrane Library Databases

| Search | Search terms used |
| --- | --- |
| Influenza (to include influenza-like conditions as well as polymerase chain reaction- or culture-confirmed influenza) | (“Influenza, Human” [MeSH], OR “influenza” OR “influenzas” OR “flu”) NOT (“Influenza in Birds” [MeSH] OR “avian influenza*” OR “bird flu”) |
| Type of study (cost, economic, or burden analyses [e.g., cost-effectiveness analysis and burden-of-illness and cost-of-illness studies]; HRQoL; absenteeism; productivity) | “Costs and Cost Analysis” [MeSH] OR “Economics, Medical” [MeSH] OR “Cost-Benefit Analysis” [MeSH] OR “Cost of Illness” [MeSH] OR “Health Care Costs” [MeSH] OR “Hospital Costs” [MeSH] OR “Economics, Hospital” [MeSH] OR “Drug Costs” [MeSH] OR (cost* OR burden OR budget OR resource*) OR (utilit* AND cost*) OR “Absenteeism” [MeSH] OR “Sick Leave” [MeSH] OR “Efficiency” [MeSH] OR “absenteeism” OR “sick leave” OR “sick day” OR efficiency OR productivity OR “Quality of Life” [MeSH] OR “quality of life” OR “quality-of-life” OR “QoL” OR “health related quality of life” OR “health-related quality-of-life” OR “HRQoL” |

HRQoL = health-related quality of life; MeSH = Medical Subject Heading.

**Supplemental File 2 - Studies reporting data not specific to culture-confirmed influenza**

| **Reference** | **Study design** | **Setting** | **Sample** | **Age** | **Study quality^a^** | **Study limitations/ bias** | **Type of data** |
| --- | --- | --- | --- | --- | --- | --- | --- |
| **Austria** | | | | | | | |
| Vutuc and Kunze, 1993[1] | Retrospective, observational study assessing hospital discharge records for the incidence of influenza diagnoses in 1990  Follow-up: 1 year | Hospital records | 3,808 patients treated for influenza | All ages considered | 2b: retrospect-tive cohort study | No specific data for children | Resource use, cost (direct) |
| **Finland** | | | | | | | |
| Järvinen et al., 2007[2] | Decision-analytic model to determine the cost-effectiveness of the use of oseltamivir compared with usual care for treatment of ILI  Time horizon: lifetime | Model considering a clinical pathway for a patient presentation to a GP with ILI | NA | Separate analyses were carried out for (1) otherwise healthy adults aged 13-64 years, (2) children aged 1-12 years, and (3) at-risk patients, aged ≥ 65 years with or without underlying comorbid-ities | 2b: cost-utility analysis | Not a population-based study | Absenteeism, cost (direct and indirect)^b^ |
| **France** | | | | | | | |
| Carrat, 2002[3] | Review that included cost data based on the Carrat et al. (2002)[4] study, as detailed in this table  Follow-up: 15 days (January-March 2000) | Physician visit for households with 1 member with fever and respiratory signs | 817 household contacts  313 developed clinical influenza | 0-90 years | 4: economic analysis | Not a population-based study; no specific data for children | Cost (direct and indirect) |
| Fagnani and German-Fattal, 2003[5] | Retrospective, longitudinal, case-control analysis to investigate costs and prescribing patterns for patients with rhinopharyngitis, tonsillitis, or an influenza-like condition treated with fusafungine, compared with the control group  Follow-up: 1 year (December 1999-November 2000) | Database of 1,010 representa-tive GPs | 44,328 with rhinopharyngitis or an ILI | All ages considered; age range was 1-108 years | 3b: retrospect-tive case-control study | Not a population-based study; no specific data for children | Resource use, cost (direct) |
| Gaillat et al., 2005[6] | Prospective, longitudinal study to describe real-life conditions for the therapeutic management of influenza and the motivations for GPs to prescribe neuraminidase inhibitors  Follow-up: 4 months (7 February 2003-30 May 2003) | General practice | 660 patients | All patients ≥ 1 year old | 2b: prospective cohort study | Not a population-based study; no specific data for children | Resource use, absenteeism |
| Mermilliod, 1975[7] | Government-sponsored feasibility study to determine the cost-savings associated with influenza vaccination of certain populations in France  Time horizon: 5 years (study conducted in 1973) | Community setting in 6 sectors of the population (as described in “sample” column) | Infants:  383,000  Elderly in care homes:  284,000  Elderly living at home:  1,910,000  Patients with chronic respiratory disease:  800,000  Pregnant women: 51,000  Active population: 386,000  Students: 3,300,000 | All ages considered | 2b: cost-of-illness analysis |  | Cost (direct and indirect) |
| **Germany** | | | | | | | |
| Szucs et al., 2001[8] | Cost-of-illness study to investigate the cost of influenza in 1996, using a costs model programmed with official aggregated statistical and publicly available data  The model used a top-down approach using the ICD-9 code 407  Follow-up: 1 year | Community: official aggregated statistical and publicly available data | Based on the whole population of Germany  4 million cases of influenza | All ages considered | 2b: economic analysis | No specific data for children | Resource use, cost (direct and indirect), absenteeism |
| **Italy** | | | | | | | |
| Colombo et al., 2001[9] | Randomised, controlled trial to compare vaccinated and non-vaccinated populations  Follow-up:  up to 6 months (December 1995-April 1996) | Healthy children recruited through paediatri-cians | 344  177 received influenza vaccine  167 received no vaccine | 1-6 years | 1b: individual randomised, controlled trial | Not a population-based study | Absenteeism |
| Esposito et al., 2003[10] | Randomised, placebo-controlled, double-blind study to evaluate effectiveness of influenza vaccination in reducing respiratory-related morbidity in children with recurrent respiratory tract infection and their families  Follow-up:  5 months (December 2000-April 2001) | Paediatric department: children with a history of recurrent respiratory tract infections | 127 children  64 received influenza vaccine  63 received placebo | 6 months to 9 years | 1b: randomised, controlled trial | Not a population-based study | Resource use, absenteeism |
| Esposito et al., 2006[11] | Prospective, randomised, single-blind, single-centre study to determine cost-savings in healthy children receiving vaccination vs. no vaccination.  Follow-up:  2003-2004 influenza season (18 weeks) | Outpatient clinic at paediatric department | 303 children  202 received 2 doses of inactivated, trivalent, virosome-formulated subunit influenza vaccine  101 received no vaccine | 2-5 years | 1b: randomised, controlled trial |  | Resource use, cost (direct and indirect), absenteeism |
| Gasparini et al., 1992[12] | Retrospective database study of patients hospitalised for respiratory illness during an influenza epidemic  Follow-up: December 1989-March 1990 | Hospitalised patients | 751 patients | All ages | 2b: retrospect-tive cohort study | Not a population-based study; no specific data for children | Resource use, cost (direct) |
| Marchetti et al., 2007[13] | Analysis to determine the incremental cost-effectiveness of using adjuvant influenza vaccination in healthy children aged 6-60 months  Time horizon of model: 5 years | Cost-effectiveness analysis | Not applicable | 6-60 months | 2b: cost-effective-ness analysis | Not a population-based study | HRQoL,  cost (direct and indirect)^b^ |
| Sauro et al., 2006[14] | Prospective, observational study to determine the increase in GPs’ workloads due to influenza  Follow-up:  5 months (November 2002-March 2003) | 5 GPs working in Caserta, Italy, observed patients with influenza, ILI, URTI, and LRTI | 328 patients with influenza  180 patients with ILI  944 patients with a URTI  739 patients with an LRTI | 1 year to > 65 years | 3b: prospective cohort study with very limited population | Not a population-based study; no specific data for children | Resource use |
| Sessa et al., 2001[15] | Prospective, observational study investigating the epidemiology and resource use associated with influenza in a general population setting  Follow-up:  3 months (December 1998-March 1999) | GPs reported the number of visits performed for ILI, clinical influenza, and any other cause during a 3-month winter epidemic period | 197,437 visits to 202 GPs for influenza or ILI, of which 6,057 cases of clinical influenza were evaluated | All ages considered | 1b: prospective cohort study with good follow-up | No specific data for children | Resource use, absenteeism |
| **The Netherlands** | | | | | | | |
| Assink et al., 2009[16] | Retrospective database analysis using data from the University of Groningen in-house prescription database and influenza surveillance data from the Dutch Working Group on Clinical Virology  Follow-up: 8 years (1998-2006) | In-house prescription database and virological surveillance | 500,000 individuals | All ages considered | 2c: audit or outcomes research | Not a population-based study | Resource use |
| Postma et al., 2007[17] | Analysis to determine the cost-effectiveness of a 5-day course of oseltamivir treatment in patients at increased risk for influenza complications  Time horizon: not presented | Cohort of patients with ILI visiting a GP | NA | > 12 years | 2b: economic analysis (limited review of the evidence) | Not a population-based study; no specific data for children | Resource use, absenteeism, cost (direct and indirect)^b^ |
| **Spain** | | | | | | | |
| Badia Llach et al., 2006[18] | Observational, longitudinal, multi-centre study of resource use associated with influenza  Follow-up:  6 months (November 1999-April 2000) | Subjects with influenza, attending 1 of the following: 2 primary care centres, 3 hospital emergency services, or the medical service at an elderly care facility | 662 patients | All ages considered | 2c: outcomes research | Not a population-based study | Resource use, cost (direct and indirect) |
| Castilla et al., 2006[19] | Retrospective, observational study to quantify the incidence of influenza and the effectiveness of the influenza vaccine  Follow-up:  2004-2005 influenza season | Community setting: network of sentinel doctors that covered a population of 22,339 inhabitants of Navarra | 22,339 inhabitants in the network | < 15 years: 32%  > 65 years: 3.8% | 2b: retrospect-tive cohort study |  | Absenteeism |
| de Juanes et al., 2006[20] | Cost-effectiveness analysis to compare a vaccination with a non-vaccination strategy  Time horizon: 1 year | Cost-effectiveness analysis | 2 theoretical cohorts (vaccinated and non-vaccinated) of 1,000 workers each | 16-65 years | 3b: economic analysis (including sensitivity analyses incorporate-ing clinically sensible variations) | Not a population-based study; no specific data for children | Resource use, absenteeism, cost (direct and indirect)^b^ |
| Gavira and Lardinois, 1990[21] | Cost-effectiveness analysis of influenza vaccine  Time horizon: 4 successive vaccination campaigns  (1984-1988) | Community: inhabitants of a rural village | 1,750 | All ages | 3b: economic analysis (including sensitivity analyses incorporate-ing clinically sensible variations) | No specific data for children | Cost (direct and indirect)^b^ |
| Navas et al., 2007[22] | Cost-effectiveness analysis to evaluate the efficiency of universal vaccination consisting of a single dose of inactivated virosomal subunit influenza vaccine compared with no vaccine  Time horizon: 6 months | Primary care centres of the Catalan Health Service | Theoretical cohort of preschool and school-aged children: N = 1,000 | 3-14 years | 3b: economic analysis (including sensitivity analyses incorporate-ing clinically sensible variations) | Not a population-based study | Resource use, absenteeism, cost (direct and indirect)^b^ |
| Pueyo Subias et al., 1994[23] | Cost-effectiveness analysis of influenza vaccine  Time horizon: 4 successive vaccination campaigns  (1984-1988) | Community: inhabitants working in the Ciudad Real area requesting disability allowance | N = 49,312, estimated to be part of the labour force in the Ciudad Real area  402 influenza-related cases requesting disability allowance on the Transitory Labour Disability programme | 15-64 years | 2b: retrospect-tive cohort study | No specific data for children | Absenteeism, cost (indirect) |
| Salleras et al., 2009[24] | Cost-benefit analysis comparing a single dose of inactivated virosomal subunit influenza vaccine (Inflexel V) with no vaccine  Time horizon: 6 months | Theoretical cohort of healthy preschool and school-aged children attending private paediatric offices in Catalonia | 1,000 children | 3-14 years | 3b: economic analysis (including sensitivity analyses incorporate-ing clinically sensible variations) | Not a population-based study | Resource use, absenteeism, cost (direct and indirect)^b^ |
| **Sweden** | | | | | | | |
| Payne et al., 2005[25] | A cross-sectional, retrospective telephone survey to determine the point prevalence of influenza in the Swedish population  Follow-up:  1 week  (14-20 February 2005) | Random sample of households: fixed-line telephone numbers were randomly sampled and households interviewed concerning influenza illness | 1,334 households called  872 households agreed to participate  2,104 individuals involved in the study | ≥ 16 years | 2b: retrospect-tive cohort study | No specific data for children | Absenteeism |
| **UK** | | | | | | | |
| Burch et al., 2009[26] | Systematic review and economic-decision modelling study to examine the clinical effectiveness and cost-effectiveness of antiviral drugs (oseltamivir and zanamivir) for the treatment of influenza  Time horizon: lifetime | Mixed: patients presenting with typical symptoms of influenza or healthy patients with experi-mentally induced influenza | Not available (literature review) | < 18 years | 1a: systematic review (with homogene-ity) of level 1 economic studies | Not a population-based study | Resource use, HRQoL,  cost (direct)^b^ |
| Jefferson et al., 2008[27] | Systematic review and meta-analysis to assess the outcomes of influenza vaccine compared with placebo or no intervention  Different follow-up periods were used in the individual studies | Literature review of randomised, controlled trials and cohort and case-control studies of influenza vaccine in healthy children | 51 studies with 294,159 observations were included  16 randomised, controlled trials and 18 cohort studies were included in the analysis of vaccine efficacy and effectiveness | < 16 years | 1a: systematic review (with homogene-ity) of randomised, controlled trials | Not a population-based study | Resource use, absenteeism |
| Meier et al., 2000[28] | Retrospective, population-based study to quantify influenza burden using the UK-based GPRD  Follow-up:  6 years (January 1991-December 1996) | Subjects registered in GPRD with at least 1 clinical diagnosis of influenza or ILI | Base population: 3,298,045  Study population: 141,293 | All ages considered | 2c: audit or outcomes research | NA | Resource use |
| Morgan et al., 2010[29] | Retrospective database analysis of routine inpatient data to determine burden of seasonal infections, including influenza, in children  Follow-up: between 2001-2002 and 2007-2008 | Children admitted to hospital with an ICD-10 code of a diagnosis relating to a selected seasonal infection | Total number of admission for the selected seasonal infections was 64,879, of which 32,126 were associated with gastrointestinal infections | ≤ 4 years | 2c: audit or outcomes research | Not a population-based study | Resource use, cost (direct) |
| Pitman et al., 2007[30] | Retrospective database analysis to determine the burden of influenza relative to GP consultations, hospitalisations, and deaths  Follow-up:  1990-2004 | National laboratory reports from the Health Protection Agency Centre for Infections, GPRD, Hospital Episode Statistics, and Office of National Statistics | NA; no specific sample size | All ages considered | 2c: audit or outcomes research |  | Resource use |
| Sander et al., 2005[31] | Analysis to determine the cost-effectiveness of treating an otherwise healthy adult population for influenza during a typical annual epidemic period  The analysis evaluated the cost-effectiveness of a course of oseltamivir and zanamivir  Time horizon:  not stated | General practice: healthy adult population | NA; cost-effectiveness model | 13-64 years | 1b: economic analysis | Not a population-based study; no specific data for children | Absenteeism, cost (direct and indirect)^b^ |
| Sander et al., 2006[32] | Study to determine the incremental cost-effectiveness and cost-utility of preventing post-exposure influenza infection with oseltamivir  The analysis evaluated the cost-effectiveness of a course of oseltamivir compared with no prophylaxis  Time horizon:  not presented | General practice: population exposed to a person with ILI within a closed environment | NA; cost-effectiveness model | ≥ 13 years | 1b: economic analysis | Not a population-based study; no specific data for children | Resource use, HRQoL,  cost (direct)^b^ |
| Turner et al., 2003[33] | Systematic review and economic-decision modelling study to examine the clinical effectiveness and cost-effectiveness of amantadine, oseltamivir, and zanamivir vs. standard care for the prevention and treatment of influenza A and B  The economic analysis evaluated the cost-effectiveness of a course of oseltamivir and zanamivir vs. standard care  Time horizon:  not presented | Literature review of clinical trials investigating amantadine, oseltamivir, and zanamivir | NA (literature review) | ≤ 12 years | 1a: systematic review (with homogene-ity) of level 1 economic studies | Not a population-based study | Resource use, HRQoL,  cost (direct)^b^ |
| **Multicountry studies** | | | | | | | |
| Paget et al., 2010[34]  (England [UK], the Netherlands, Italy, and Spain) | European Paediatric Influenza Analysis prospective study in which data were collected from surveillance networks on the rates of consultation for influenza in children aged 0-14 years  Follow-up:  2002-2008 | Surveillance networks containing data on existing weekly virological and age-specific ILI | England: approximately 500 GPs covering 1.8% of the population  Italy: 762 sentinel GPs and 213 paediatricians, covering 2% of the population  The Netherlands: approximately 65 GPs covering about 1% of the population  Spain: approximately 497 GPs and 171 paediatricians covering approximately 2% of the population | ≤ 14 years | 2b: individual cohort study | Not a population-based study | Resource use |
| Ryan et al., 2006[35]^c^  25 countries in the EU (including Austria, Finland, France, Germany, Italy, the Netherlands, Spain, Sweden, and the UK) | Model to determine how many individuals are eligible for influenza vaccination and to estimate the cost and consequence of not vaccinating this population for 5 EU member states: France, Germany, Italy, Spain, and the UK  Time horizon: duration of interpandemic periods | Data sources included: 2005 population projects from the World Bank, The Global Initiative for Asthma, WHO figures, the Spanish National Organisation of Transplants, and the European Health for All Database | 223,442,807  Austria: 3,671,215  Finland: 2,670,973  France: 29,248,037  Germany: 40,906,249  Italy: 29,440,014  Netherlands: 7,747,467  Spain: 18,265,242  Sweden: 4,889,950  The UK: 33,464,844  Risk groups constituted ≤ 50% of overall population | At-risk populations (aged 0-4 years, aged 50-64 years, aged ≥ 65 years, other [health care workers or respiratory disease, diabetes, cardio-vascular disease, HIV/AIDS, or transplant patients]) | 3b: economic analysis (including sensitivity analyses incorporate-ing clinically sensible variations) | Not a population-based study; no specific data for children; no specific data for countries of interest | Resource use |
| Ashkenazi et al., 2006[36]  9 European countries (Belgium, Czech Republic, Finland. Germany, Italy, Poland, Spain, Switzerland, and the UK) | Randomised, open-label study to compare CAIV-T and TIV in children  Follow-up:  8 months (October 2002-June 2003) | Children with a history of recurrent respiratory tract infections | 2,187 patients  1,101 receiving CAIV-T  1,086 receiving TIV | 6-71 months | 1b: randomised, controlled trial | No specific data for countries of interest | Resource use, absenteeism |
| Fleming et al., 2006[37]  13 European countries (Belgium, Finland, Germany, Greece, Israel, Italy, the Netherlands, Norway, Poland, Portugal, Spain, Switzerland, and the UK) | Randomised, open-label, active-controlled trial to compare CAIV-T with TIV in children  Follow-up: 8 months (October 2002-May 2003) | Children with a clinical diagnosis of asthma | 2,229 patients  1,114 receiving CAIV-T  1,115 receiving TIV | 6-17 years | 1b: randomised, controlled trial | No specific data for countries of interest | Resource use, absenteeism |
| Vesikari et al., 2006[38]  5 European countries (Belgium, Finland, Israel, Spain, and the UK) | Randomised, double-blind, placebo-controlled trial to compare CAIV-T with placebo in children  Follow-up: 20 months (October 2000-May 2002) | Community: children attending day care | Year 1:  1,616 patients (951 receiving vaccine; 665 receiving placebo)  Year 2:  1,090 patients (640 receiving vaccine; 450 receiving placebo) | 6 to < 36 months | 1b: randomised, controlled trial | No specific data for countries of interest | Resource use, absenteeism |

AIDS = acquired immune deficiency syndrome; CAIV-T = cold-adapted influenza vaccine, trivalent; EU = European Union; GP = general practitioner; GPRD = General Practice Research Database; HIV = human immunodeficiency virus; HRQoL = health-related quality of life; ICD-9 = *International Classification of Diseases, 9th Revision*; ICD-10 = *International Classification of Diseases, 10th Revision*; ILI = influenza-like illness; LRTI = lower respiratory tract infection; NA = not applicable; TIV = trivalent influenza vaccine; UK = United Kingdom; URTI = upper respiratory tract infection; WHO = World Health Organization.

Note: Most studies (except one) reported data for ILI and therefore were not specific to influenza. One exception study [38] reported data on culture-confirmed influenza cumulatively for children in the treatment and the placebo groups. Although these estimates, coming from a randomised, controlled trial, did reflect the burden of influenza, they were not specific to only patients with culture-confirmed influenza.

^a^ Study quality according to the Centre for Evidence Based Medicine scale [39].

^b^ Cost-effectiveness study.

^c^ Resource use data were estimated using a mathematical model programmed with vaccine distribution data, health economics data, and demographic data on populations at risk for influenza for 25 countries within the EU.

**Supplemental File 3 - Health care resource use and absenteeism in children with confirmed influenza**

| **Reference, country, study period** | **Population (n)** | **Health care resource use** | **Absenteeism** |
| --- | --- | --- | --- |
| Heikkinen et al., 2004[40]  Finland  Influenza seasons: 2000-2001 (follow-up: 7.5 months), 2001-2002 (follow-up: 7.5 months) | 2,231 children aged ≤ 13 years  372 (16.7%) had culture-confirmed influenza  Setting: community (day care, family day care, schools) | **Resource use for all children (n = 370), children aged < 3 years (n = 131), children aged 3-6 years (n = 148), and children aged 7-13 years (n = 91), both seasons combined**  GP consultation and hospitalisation:  Referral to ED: 0.8%; 2.3%; 0%; 0%  Hospitalisation: 0.3%; 0.8%; 0%; 0%  Visits to GP: NR  Medication:  Antibiotic treatment: 28.1%; 42.0%; 27.7%; 8.8% | **Children aged < 3 years (n = 103), children aged 3-6 years (n = 128), and children aged 7-13 years (n = 76), both seasons combined**  Children’s absence from day care or school:  Absent for ≥ 1 day: 75.7%; 73.4%; 77.6%  Mean duration of absence: 3.6 days (SE: 0.2 days); 3.6 days (SE: 0.2 days); 2.8 days (SE: 0.2 days)  Mean duration of absence regardless of age; 3.4 days^a^  Total days of absence per 100 children with influenza: 274 days (95% CI: 227-320 days); 262 days (95% CI: 224-300 days); 216 days (95% CI: 175-257 days)  Parents’ absence from work:  Absent for ≥ 1 day: 61.2%; 53.9%; 26.3%  Total average percentage of parents absent from work (regardless of child’s age): 49.4%^b^  Mean duration of absence: 3.2 days (SE: 0.2 days); 2.7 days (SE: 0.2 days); 2.1 days (SE 0.3 days)  Total days of absence per 100 children with influenza: 195 days (95% CI: 153-238 days; 146 days (95% CI: 117-176 days); 54 days (95% CI: 28-80 days)  Mean duration of absence regardless of age; 2.7 days^b^ |
| Heinonen et al., 2010[41]  Finland  Influenza season:  2007-2009 (follow-up: 21 days) | 408 patients aged 1-3 years with ILI  Patients with confirmed influenza: 98 (24.7%), including 61 receiving placebo, and 37 receiving oseltamivir  Setting: paediatric clinic | **Mean number of doses of antipyretics/antibiotics, 21-day follow-up (NR):**  All confirmed cases: 5.9  Influenza A: 6.1  Influenza B: 5.1 | **Child’s absence from day care for group receiving placebo (median days [IQR]), 21-day follow-up:**  All influenza (n = 61): 4.0 (3.0-5.0)  Influenza A (n = 50): 4.0 (3.0-5.0)  Influenza B (n = 11): 1.5 (1.0-4.0)  **Parent’s absence from work for group receiving placebo (median days [IQR]), 21-day follow-up:**  All influenza (n = 61): 2.0 (0.0-4.0)  Influenza A (n = 50): 3.0 (0.0-4.0)  Influenza B (n = 11): 1.0 (0.0-3.0) |
| Ploin et al., 2003[42]  France  Influenza season: January-February 2002  (follow-up: 15 days) | 304 children aged 0-11 months  Setting: paediatric ED  Children with virology-confirmed influenza: 99 (33%)  Follow-up interviews conducted on days 8 and 15 after the ED visit | **Proportion of patients in each group who were hospitalised:**  Influenza positive (n = 99): 20%  Influenza negative (n = 205): 19%  Influenza A (n = 90): 20%  Influenza B (n = 9): 22% (*P* = 0.87 vs. influenza A)  **Resource use in children with confirmed influenza:**  Hospitalisation and medical visits:  Mean length of hospital stay: 7.9 days  Mean number of medical visits: 2.8  GP visits: NR (because all visits were to the ED)  Medication and therapeutic care:  Physiotherapy visits: 14%  Prescribed symptomatic treatment: 78%  Antibiotic treatment: 42%  Diagnostic care:  Blood tests: 22%  Roentgenograms: 10%  Urinary tests: 26% | Major change in day care in children with confirmed influenza (n = 92): 26%  Parent’s absence for children with confirmed influenza (n = 92): 53% |
| Ploin et al., 2007[43]  France  Influenza season: January-February 2002  (follow-up: 15 days) | 575 children aged < 36 months, with ILI  283 (49%) children had ELISA-confirmed influenza  Setting: paediatric ED | **Resource use in children with positive (n = 263) and negative (n = 275) influenza-virus detection, 15 days’ follow-up**  GP consultation and hospitalisation:  Hospitalisations: 10%; 18%  **Hospitalisations in patients with influenza A (n = 253) and B (n = 30): 9% and 13%, respectively (*P* = 0.41):**  Mean (SD) length of stay: 1.8 days (1.3 days); 2.7 days (2.1 days)  Extra medical visits (SD): 70% or 2.1 visits per child (1.4 visits); 69% or 2.1 visits per child (1.5 visits)  Medication and therapeutic care:  Respiratory physiotherapy visits (SD): 10% or 6.0 visits per child (3.7 visits); 9% or 7.8 visits per child (6.8 visits)  Prescribed symptomatic treatment: 86%; 83%  Prescribed antibiotic treatment: 42%; 28%  Diagnostic procedures:  Blood tests: 14%; 15%  Roentgenogram: 11%; 10%  Urinary tests: 15%; 14% | **Parent’s absence, 15 days’ follow-up**  Positive influenza-virus detection (n = 263): 54%  Mean (SD) length: 6.3 (4.7) days  Negative influenza-virus detection (n = 275): 54%  Mean (SD) length: 5.9 (4.3) days |
| Sanni et al., 2004[44]  France  Influenza season: January-February 2002  (follow-up: 5.5 weeks) | 472 children with ILI aged ≤ 15 years  Children with laboratory-confirmed influenza: 59 (51.8%) of 114 tested  Setting: paediatric hospital | **Hospitalisation (length of stay in children with confirmed influenza) (n = 59), 5.5 weeks’ follow-up:**  Median (range) hospital stay: 3 (1-10) days  Mean stay in general paediatric care: 4 days  **Medication (antibiotics in children with confirmed influenza), 5.5 weeks’ follow-up:**  Total (n = 59): 33.9%  Aged < 1 years (n = 14): 42.9%  Aged > 1 to ≤ 3 years (n = 31): 41.9%  Aged > 3 years (n = 14): 7.1%  Note: No effect size data available | NR |
| Bosis et al., 2005[45]  Italy  Influenza season: November 2002-March 2003  (follow-up: 5 months) | 1,505 children aged < 15 years  Setting: ED  Of these, 1,019 children with acute respiratory infection  Influenza was confirmed by PCR in 230 (15.3%) of total cases | **Resource use in children with confirmed influenza (n = 223), 5 months’ follow-up:**  Diagnostic procedures:  Routine blood examinations: 4.5%  Microbiological tests: 3.6%  Chest radiography: 8.9%  GP consultation and hospitalisation:  Hospitalisation: 5.4%  Medical visits: NR  Medication:  Antipyretic prescriptions: 77.6%; *P* < 0.0001 vs. RSV-positive children  Antibiotic prescriptions: 52.0%  Bronchodilator prescriptions: 13.5%  Steroid prescriptions: 11.2%  **Resource use in families of children with confirmed influenza (n = 806), 5 months’ follow-up:**  GP consultation and hospitalisation:  Additional medical visits: 9.7%  Hospitalisation: 0.4%  Medication:  Antipyretic prescriptions: 12.9%  Antibiotic prescriptions: 4.5% | **Data from 5 months’ follow-up:**  Median (range) school absence in children with confirmed influenza (n = 223): 12 (5-15) days  Median work days lost in families of the study children (n = 806): 4 (1-10) days (*P* < 0.05)  Median school days lost in families of children with confirmed influenza (n = 806): 5 (1-15) days (*P* < 0.05) |
| Esposito et al., 2005[46]  Italy  Influenza season: November 2002-March 2003  (follow-up: 5 months) | 1,520 children aged < 15 years, with PCR-confirmed influenza or RSV  Setting: ED  Influenza confirmed in 234 (15.4%) children | **Resource use in children with confirmed influenza (n = 234), 5 months’ follow-up:**  Hospitalisation: 5.6%  Medication:  Bronchodilators: 6.4%  Steroids: 11.5%  Antipyretic treatment: 82.5%  Diagnostic procedures:  Microbiological diagnostic tests: 2.1%  **Resource use in families of influenza-positive children (n = 651), 5 months’ follow-up:**  GP consultation and hospitalisation:  Hospitalisation: 0.3%  Additional medical visits: 13.5%  Medication:  Antipyretic prescriptions: 16.4%  Antibiotic prescriptions: 5.4% | **Data from 5 months’ follow-up:**  Median school absence in children with confirmed influenza (n = 234): 12 days  Days lost due to influenza in families of the influenza-positive children (n = 651)  Median (range) work days missed by mothers: 4 (1-9) days  Median work days missed by fathers:  3 (2-8) days  Median school days missed: 6 (2-15) days |
| Esposito et al., 2011[47]  Italy  Influenza season: November 2008-April 2009 | Of 21,986 children aged < 14 years followed up by 50 primary care physicians, 6,988 (31.8%) presented with signs or symptoms of ILI  PCR-confirmed influenza in 2,143 (30.7%) cases  Setting: paediatric primary care | **Resource use in influenza-negative (n = 4,845) and influenza-positive children (n = 2,143), until resolution of illness:**  ED visit: 2.1%; 2.6%  Hospitalisation: 0.8%; 0.7%  Antibiotic prescriptions: 52.0%; 43.0%  Antipyretic prescriptions: 93.1%; 99.3%  Mean (SD) duration of antipyretic: 2.9 days (2.34 days), *P* < 0.05; 3.9 days (3.5 days)  Further examinations: 15.0%; 23.9% (*P* < 0.001)  **Resource use in influenza-positive children (influenza A [n = 1,751]; influenza B [n = 392]), until resolution of illness:**  ED visit: 2.8%; 1.5%  Hospitalisation: 0.8%; 0.5%  Antibiotic prescriptions: 43.7%; 39.7%  Antipyretic prescriptions: 99.9%; 97.2%  Mean (SD) duration of antipyretic: 4.1 days (3.4 days); 3.4 days (3.1 days)  Further examinations: 25.2%; 18.6% (*P* < 0.05)  **Resource use in influenza-positive children (aged < 2 years [n = 343]; 2-5 years [n = 1,071]; > 5 years [n = 729]), until resolution of illness:**  ED visit: 2.9%; 3.1%; 1.6%  Hospitalisation: 1.5%; 0.7%; 0.4%  Antibiotic prescriptions: 37.9% (*P* < 0.05 vs. 2-5 years); 47.3%: 39.0% (*P* < 0.05 vs. 2-5 years)  Antipyretic prescriptions: 99.1%; 99.4%; 99.5%  Mean (SD) duration of antipyretic: 4.4 days (3.2 days); 3.9 days (3.6 days); 3.6 days (4.1 days)  Further examinations: 25.9%; 23.3%; 24.0% | **Absenteeism of parents in influenza-negative (n = 4,845) and influenza-positive children (n = 2,143) in the 7 days following the child’s diagnosis:**  Similar disease in household: 25.0%; 43.0%; *P* < 0.001  Mothers absent from work: 12.0%; 16.3%; *P* < 0.001  Mean (SD) working days lost by mothers: 3.39 days (2.26 days); 4.46 days (2.11 days); *P* < 0.05  Fathers absent from work: 2.0%; 6.1%; *P* < 0.05  Mean (SD) working days lost by fathers: 1.96 days (2.04 days); 4.31 days (2.73 days); *P* < 0.001  Parental absenteeism across all age groups (for both mothers and fathers): 11%^c^  **Absenteeism of parents in influenza-positive children with influenza A (n = 1,751) or influenza B (n = 392); follow-up not reported:**  Similar disease in household: 45.2%; 33.4%; *P* < 0.001  Mothers absent from work: 18.0%; 8.4%; *P* < 0.05  Mean (SD) working days lost by mothers: 4.57 days (2.43 days); 2.99 days (1.90 days); *P* < 0.05  Fathers absent from work: 7.0%; 2.0%; *P* < 0.05  Mean (SD) working days lost by fathers: 4.41 days (3.10 days); 3.00 days (2.71 days); *P* < 0.05  **Absenteeism of parents in influenza-positive children (aged < 2 years [n = 343]; 2-5 years [n = 1,071]; > 5 years [n = 729]) in the 7 days following a child’s diagnosis:**  Similar disease in household: 37.9% (*P* < 0.05 vs. 2-5 years); 51.8%; 32.5% (*P* < 0.05 vs. 2-5 years);  Mothers absent from work: 14.3%; 17.3%; 15.8%  Mean (SD) working days lost by mothers: 4.95 days (2.61 days), *P* < 0.05 vs. > 5years; 4.88 days (2.03 days), *P* < 0.05 vs. > 5years; 1.91 days (2.34 days)  Fathers absent from work: 5.5% (*P* < 0.05 vs. > 5years); 6.9% (*P* < 0.05 vs. > 5years); 2.5%  Mean (SD) working days lost by fathers: 5.61 days (2.64 days); *P* < 0.05 vs. > 5years; 4.99 days (2.88 days); *P* < 0.05 vs. > 5 years; 1.98 days (2.06 days) |
| Principi et al., 2003[48]  Italy  Influenza season: November 2001-April 2002  (follow-up: 6 months) | 3,771 children aged < 14 years, with ILI presenting to an ED or primary care physicians  352 (9.3%) had confirmed influenza  Setting: ED or primary care | **Resource use: children with positive influenza-virus detection (n = 352) and negative virus detection (n = 3,419); 6 months’ follow-up:**  GP consultation and hospitalisation:  Hospital admission: 3.9%; 5.1%  Length of hospital stay (SD): 4.08 days (1.61 days); 4.67 days (2.16 days)  Extra medical visits (SD): 0.68 (1.23); 0.66 (1.01)  **Impact of influenza in families: children with positive virus detection (n = 915 household contacts) and children with negative virus detection (n = 9,128 household contacts); 6 months’ follow-up:**  GP consultation and hospitalisation:  Hospitalisation: 0.3%; 0.1%  Number of extra medical visits (SD): 0.39 (0.76); 0.14 (0.47); *P* < 0.0001 vs. influenza-negative children | **Mean (SD) school absence; 6 months’ follow-up**  Influenza positive: 5.10 (2.55) days  Influenza negative: 4.25 (2.93) days (*P* < 0.0001)  **Impact of influenza: families of children with positive influenza-virus detection (n = 915) and contacts of children with negative influenza-virus detection (n = 9,128); 6 months’ follow-up:**  Mean (SD) lost working days by parents: influenza-positive: 1.39 days (3.09 days); influenza-negative: 0.59 days (2.02 days); *P* < 0.0001  Mean (SD) lost school days by siblings: influenza-positive: 1.27 days (2.47 days); influenza-negative: 0.49 days (2.33 days); *P* < 0.0001  Mean (SD) help needed to care for ill children: influenza-positive: 1.10 days (1.76 days); influenza-negative: 0.85 days (1.63 days); *P* < 0.0001  Mean (SD) help needed to care for ill children: influenza A: 0.77 days (1.82 days); influenza B: 1.30 days (2.35 days); *P* = 0.0016 |
| Principi et al., 2004[49]  Italy  Influenza season: November 2001-April 2002  (follow-up: 6 months) | 3,771 children aged < 14 years, with ILI  Setting: ED or primary care | **Resource use in influenza-positive (n = 352) [influenza-negative children (n = 3,149)], until resolution of illness:**  GP consultation and hospitalisation:  Hospitalisation: 3.9% [5.1%]  Mean (SD) duration of hospitalisation: 4.08 days (1.61 days) [4.67 days (2.16 days)]  Mean (SD) number of extra medical visits: 0.68 (1.23) [0.66 (1.01)]  Medication:  Antipyretic prescriptions; 76.4%; *P* < 0.0001 [59.9%]  Mean (SD) antipyretic use: 3.23 days (1.44 days), *P* < 0.0001 [2.81 days (1.45 days)]  Antibiotic prescriptions: 54.8% [56.9%]  Mean (SD) antibiotic use: 7.72 days (2.47 days) [7.71 days (2.45 days)]  Diagnostic procedures:  Routine blood examinations: 8.5% [8.9%]  Microbiological tests: 4.2% [4.9%]  Chest radiography: 4.2% [6.2%]  **Resource use: families of influenza-positive (n = 915) and influenza-negative children (n = 9,128), until resolution of child’s illness:**  Antipyretic prescriptions: 13.4%; *P* < 0.0001 [6.7%]  Antibiotic prescriptions: 7.9%; *P* < 0.0001 [3.3%]  Hospitalisation: 0.3% [0.1%]  Mean number of extra medical visits: 0.39; *P* < 0.0001 [0.14] | **Mean (SD) school absence in children, until resolution of illness:**  Influenza-positive virus detection (n = 352): 5.10 (2.55) days (*P* < 0.0001)  Influenza-negative virus detection (n = 3,419): 4.25 (2.93) days  **Number of days lost due to influenza: families of influenza-positive children (n = 915) and contacts of influenza-negative children (n = 9,128), until resolution of child’s illness:**  Mean (SD) work days lost by parents due to their own illness: 1.52 days (3.19 days), *P* < 0.0001; 0.72 days (2.14 days)  Mean (SD) work days lost by parents due to their child’s illness: 1.25 days (2.99 days), *P* < 0.0001; 0.59 days (2.02 days)  Mean (SD) school days lost by siblings: 1.27 days (2.47 days), *P* < 0.0001; 0.49 days (2.33 days)  Mean (SD) need for help to care for ill children: 1.10 days (1.76 days), *P* < 0.0001; 0.85 days (1.63 days)  Influenza A: 0.77 days (1.82 days), *P* = 0.0016  Influenza B: 1.30 days (2.35 days) |
| Van Der Zalm, 2009[50]  The Netherlands  Influenza season:  2003-2006 (follow-up: 1 year) | 305 healthy infants aged 2-3 weeks were followed up until they reached aged 1 year  Total sample: 668  Episodes of respiratory illness per child per year: 5.0  Sample with a single virus: 468  Sample with influenza virus: 8 (1.7%)  Setting: community (birth-cohort study) | Physician visits in patients with influenza-virus detected (n = 8), 1-year follow-up: 12.5%  Note: No effect size data available | NR |

CI = confidence interval; ED = emergency department; ELISA = enzyme-linked immunosorbent assay; GP = general practitioner; ILI = influenza-like illness; IQR = interquartile range; NR = not recorded; PCR = polymerase chain reaction; RSV = respiratory syncytial virus; SD = standard deviation; SE = standard error.

Note: For further details on study design and patient characteristics, see Table 1.

^a^ We calculated this value from the article as follows: (103 × 3.6 + 128 × 3.6 + 76 × 2.8) [total number of days absent by all children combined] ÷ (103 + 128 + 76) [total number of children absent] = 3.4 days [average duration of absenteeism per child].

^b^ We calculated this value from the article as follows: (103 × 3.2 + 128 × 2.7 + 76 × 2.1) [total number of days absent by all children combined] ÷ (103 + 128 + 76) [total number of children absent] = 3.4 days [average duration of absenteeism per child].

^c^ We calculated this figure from the paper as follows: 349 [mothers absent] ÷ 16.3% [percentage of mothers absent] = 2,141 [total number of mothers]; 130 [fathers absent] ÷ 6.1% [percentage of fathers absent] = 2,131 [total number of fathers]; 2,141 + 2,131 = 4,272 [total number of parents]; (349 + 130) ÷ 4,272 × 100% = 11.2% [percentage of parents absent].

**References for Online Appendix**

1. Vutuc C, Kunze M: **Influenza: incidence and costs of inpatient treatment**. *Fortschr Med* 1993, **111**:508-509.
2. Järvinen A, Joutseno J, Gyldmark M: C**ost effectiveness of oseltamivir for the treatment of influenza in adults, adolescents and children in Finland (provisional abstract)**. *J Med Econ* 2007, **10**:199-214.
3. Carrat F. **The impact of influenza on mortality and cost**. *Virologie* 2002, **6**:S97-S104.
4. Carrat F, Sahler C, Rogez S, Leruez-Ville M, Freymuth F, Le Gales C, Bungener M, Housset B, Nicolas M, Rouzioux C: **Influenza burden of illness: estimates from a national prospective survey of household contacts in France**. *Arch Intern Med* 2002, **162**:1842-1848.
5. Fagnani F, German-Fattal M: **Antibiotic prescribing patterns of French GPs for upper respiratory tract infections: impact of fusafungine on rates of prescription of systemic antibiotics**. *Am J Respir Med* 2003, **2**:491-498.
6. Gaillat J, Pecking M, El Sawi A, Grandmottet G, Schlemmer C, Barbaza MO, Carrat F: **Neuraminidase inhibitors in the general practice management of influenza: who prescribe them, when and with which results?** *Med Mal Infect* 2005, **35**:435-442.
7. Mermilliod C: **Sectorial study: influenza vaccination**. *Cah Sociol Demogr Med* 1975, **15**:52-59.
8. Szucs T, Behrens M, Volmer T: C**osts of influenza in Germany 1996: a cost-of-illness study**. *Med Klin* 2001, **96**:63-70.
9. Colombo C, Argiolas L, La Vecchia C, Negri E, Meloni G, Meloni T: **Influenza vaccine in healthy preschool children**. *Rev Epidemiol Sante Publique* 2001, **49**:157-162.
10. Esposito S, Marchisio P, Cavagna R, Gironi S, Bosis S, Lambertini L, Droghetti R, Principi N: **Effectiveness of influenza vaccination of children with recurrent respiratory tract infections in reducing respiratory-related morbidity within the households**. *Vaccine* 2003, **21**:3162-3168.
11. Esposito S, Marchisio P, Bosis S, Lambertini L, Claut L, Faelli N, Bianchi C, Colombo GL, Principi N: **Clinical and economic impact of influenza vaccination on healthy children aged 2-5 years**. *Vaccine* 2006, **24**:629-635.
12. Gasparini R, Pozzi T, Giotti M, Fatighenti D: **Excess hospitalization for respiratory illnesses during influenza epidemics in Siena between 1987 and 1990**. *J Prev Med Hyg* 1992, **33**:107-110.
13. Marchetti M, Kuhnel UM, Colombo GL, Esposito S, Principi N: **Cost-effectiveness of adjuvanted influenza vaccination of healthy children 6 to 60 months of age**. *Hum Vaccin* 2007, **3**:14-22.
14. Sauro A, Barone F, Blasio G, Russo L, Santillo L: **Do influenza and acute respiratory infective diseases weigh heavily on general practitioners’ daily practice?** *Eur J Gen Pract* 2006, **12**:34-36.
15. Sessa A, Costa B, Bamfi F, Bettoncelli G, D’Ambrosio G: **The incidence, natural history and associated outcomes of influenza-like illness and clinical influenza in Italy**. *Fam Pract* 2001, **18**:629-634.
16. Assink MD, Kiewiet JP, Rozenbaum MH, Van den Berg PB, Hak E, Buskens EJ, Wilschut JC, Kroes AC, Postma MJ: **Excess drug prescriptions during influenza and RSV seasons in the Netherlands: potential implications for extended influenza vaccination**. *Vaccine* 2009, **27**:1119-1126.
17. Postma MJ, Novak A, Scheijbeler HW, Gyldmark M, van Genugten ML, Wilschut JC: **Cost effectiveness of oseltamivir treatment for patients with influenza-like illness who are at increased risk for serious complications of influenza: illustration for the Netherlands**. *Pharmacoeconomics* 2007, **25**:497-509.
18. Badia Llach, X, Roset GM, Frances Tudel JM, Alvarez SC, Rubio TC: **Study of flu costs**. *Aten Primaria* 2006, **38**:260-267.
19. Castilla J, Arregui L, Baleztena J, Barricarte A, Brugos A, Carpintero M, Cortés F, Chérrez C, Díez J, Fernández-Alonso M, Figuerido E, Franco T, Gil A, Guijarro JL, Iceta A, Lacalle MT, Martín C, Martínez Mazo MD, Morán J, Moreno M, Palau J, Pérez-Afonso F, Rodríguez Macías A, Ruiz I, Senosiain MA, Sota M, Virto T, Vizcay JM, Yoldi C, Zubicoa J; Red Centinela de Gripe de Navarra: **Incidence of influenza and influenza vaccine effectiveness in the 2004-2005 season**. *An Sist Sanit Navar* 2006, **29**:97-106.
20. de Juanes, Jr., Cisterna R, Sanz J, Magaz S, Badia X: **Efficiency of influenza vaccination in the working population in Spain**. *Gac Sanit* 2006, **20**:101-107.
21. Gavira FJ, Lardinois R: **Cost-effectiveness analysis of antigrippal vaccination in a rural population (La Victoria, Cordoba)**. *Med Clin (Barc)* 1990, **94**:777-781.
22. Navas E, Salleras L, Dominguez A, Ibáñez D, Prat A, Sentís J, Garrido P: **Cost-effectiveness analysis of inactivated virosomal subunit influenza vaccination in children aged 3-14 years from the provider and societal perspectives**. *Vaccine* 2007, **25**:3233-3239.
23. Pueyo Subias P, Garcia Rivas JV, Barra GC, Suarez Carrillo JM: **Disability leave due to influenza in the area of Ciudad Real**. *Rev Sanid Hig Publica (Madr)* 1994, **68**:221-223.
24. Salleras L, Navas E, Dominguez A, Ibáñez D, Prat A, Garrido P, Asenjo MA, Torner N: **Economic benefits for the family of inactivated subunit virosomal influenza vaccination of healthy children aged 3-14 years during the annual health examination in private paediatric offices**. *Vaccine* 2009, **27**:3454-3458.
25. Payne L, Kuhlmann-Berenzon S, Ekdahl K, Giesecke J, Hogberg L, Penttinen P: **“Did you have flu last week?” A telephone survey to estimate a point prevalence of influenza in the Swedish population**. *Eur Surveill* 2005, **10**:241-244.
26. Burch J, Paulden M, Conti S, Stock C, Corbett M, Welton NJ, Ades AE, Sutton A, Cooper N, Elliot AJ, Nicholson K, Duffy S, McKenna C, Stewart L, Westwood M, Palmer S: **Antiviral drugs for the treatment of influenza: a systematic review and economic evaluation**. *Health Technol Assess* 2009, **13**:1-265, iii-iv.
27. Jefferson T, Rivetti A, Harnden A, Di Pietrantonj C, Demicheli V: V**accines for preventing influenza in healthy children**. *Cochrane Database Syst Rev* 2008, **2**. Article No. CD004879. DOI: 10.1002/14651858.CD004879.pub3.
28. Meier CR, Napalkov PN, Wegmuller Y, Jefferson T, Jick H: **Population-based study on incidence, risk factors, clinical complications and drug utilisation associated with influenza in the United Kingdom**. *Eur J Clin Microbiol Infect Dis* 2000, **19**:834-842.
29. Morgan C, Adlard N, Carroll S, Parvataneni L: **Burden on UK secondary care of rotavirus disease and seasonal infections in children**. *Curr Med Res Opin* 2010, **26**:2449-2455.
30. Pitman RJ, Melegaro A, Gelb D, Siddiqui MR, Gay NJ, Edmunds WJ: **Assessing the burden of influenza and other respiratory infections in England and Wales**. *J Infect* 2007, **54**:530-538.
31. Sander B, Gyldmark M, Hayden FG, Morris J, Mueller E, Bergemann R: **Influenza treatment with neuraminidase inhibitors: cost-effectiveness and cost-utility in healthy adults in the United Kingdom**. *Eur J Health Econ* 2005, **6**:244-252.
32. Sander B, Hayden FG, Gyldmark M, Garrison LP Jr: **Post-exposure influenza prophylaxis with oseltamivir: cost effectiveness and cost utility in families in the UK**. *Pharmacoeconomics* 2006, **24**:373-386.
33. Turner D, Wailoo A, Nicholson K, Cooper N, Sutton A, Abrams K: **Systematic review and economic decision modelling for the prevention and treatment of influenza A and B**. *Health Technol Assess* 2003, **7**.
34. Paget WJ, Balderston C, Casas I, Donker G, Edelman L, Fleming D, Larrauri A, Meijer A, Puzelli S, Rizzo C, Simonsen L; EPIA Collaborators: **Assessing the burden of paediatric influenza in Europe: the European Paediatric Influenza Analysis (EPIA) project**. *Eur J Pediatr* 2010, **169**:997-1008.
35. Ryan J, Zoellner Y, Gradl B, Palache B, Medema J: **Establishing the health and economic impact of influenza vaccination within the European Union 25 countries**. *Vaccine* 2006, **24**:6812-6822.
36. Ashkenazi S, Vertruyen A, Arístegui J, Esposito S, McKeith DD, Klemola T, Biolek J, Kühr J, Bujnowski T, Desgrandchamps D, Cheng SM, Skinner J, Gruber WC, Forrest BD; CAIV-T Study Group: **Superior relative efficacy of live attenuated influenza vaccine compared with inactivated influenza vaccine in young children with recurrent respiratory tract infections**. *Pediatr Infect Dis J* 2006, **25**(10):870-879.
37. Fleming DM, Crovari P, Wahn U, Klemola T, Schlesinger Y, Langussis A, Øymar K, Garcia ML, Krygier A, Costa H, Heininger U, Pregaldien JL, Cheng SM, Skinner J, Razmpour A, Saville M, Gruber WC, Forrest B; CAIV-T Asthma Study Group. **Comparison of the efficacy and safety of live attenuated cold-adapted influenza vaccine, trivalent, with trivalent inactivated influenza virus vaccine in children and adolescents with asthma**. *Pediatr Infect Dis J* 2006, **25**(10):860-869.
38. Vesikari T, Fleming DM, Aristegui JF, Vertruyen A, Ashkenazi S, Rappaport R, Skinner J, Saville MK, Gruber WC, Forrest BD; CAIV-T Pediatric Day Care Clinical Trial Network. Safety, efficacy, and effectiveness of cold-adapted influenza vaccine-trivalent against community-acquired, culture-confirmed influenza in young children attending day care. *Pediatrics* 2006, **118**(6):2298-2312.
39. Oxford Centre for Evidence-based Medicine: **Levels of Evidence** [http://www.cebm.net/index.aspx?o=1025]
40. Heikkinen T, Silvennoinen H, Peltola V, Ziegler T, Vainionpaa R, Vuorinen T, Kainulainen L, Puhakka T, Jartti T, Toikka P, Lehtinen P, Routi T, Juven T: **Burden of influenza in children in the community**. *J Infect Dis* 2004, **190**:1369-1373.
41. Heinonen S, Silvennoinen H, Lehtinen P, Vainionpää R, Vahlberg T, Ziegler T, Ikonen N, Puhakka T, Heikkinen T: **Early oseltamivir treatment of influenza in children 1-3 years of age: a randomized controlled trial**. *Clin Infect Dis* 2010, **51**:887-894.
42. Ploin D, Liberas S, Thouvenot D, Fouilhoux A, Gillet Y, Denis A, Chapuis F, Lina B, Floret D: **Influenza burden in children newborn to eleven months of age in a pediatric emergency department during the peak of an influenza epidemic**. *Pediatr Infect Dis J* 2003, **22**(Suppl 10):S218-S222.
43. Ploin D, Gillet Y, Morfin F, Fouilhoux A, Billaud G, Liberas S, Denis A, Thouvenot D, Fritzell B, Lina B, Floret D: **Influenza burden in febrile infants and young children in a pediatric emergency department**. *Pediatr Infect Dis J* 2007, **26**:142-147.
44. Sanni E, Mazaud S, Odievre MH, Weill C, Laurent C, Olivier C: **Influenzae infection in hospitalized children**. *J Pédiatr Puér* 2004, **17**:331-337.
45. Bosis S, Esposito S, Niesters HG, Crovari P, Osterhaus AD, Principi N: **Impact of human metapneumovirus in childhood: comparison with respiratory syncytial virus and influenza viruses**. *J Med Virol* 2005, **75**:101-104.
46. Esposito S, Gasparini R, Bosis S, Marchisio P, Tagliabue C, Tosi S, Bianchi C, Crovari P, Principi N: **Clinical and socio-economic impact of influenza and respiratory syncytial virus infection on healthy children and their households**. *Clin Microbiol Infect* 2005, **11**:933-936.
47. Esposito S, Cantarutti L, Molteni CG, Daleno C, Scala A, Tagliabue C, Pelucchi C, Giaquinto C, Principi N: **Clinical manifestations and socio-economic impact of influenza among healthy children in the community**. *J Infect* 2011, **62**:379-387.
48. Principi N, Esposito S, Marchisio P, Gasparini R, Crovari P: **Socioeconomic impact of influenza on healthy children and their families**. *Pediatr Infect Dis J* 2003, **22**(Suppl 10):S207-S210.
49. Principi N, Esposito S, Gasparini R, Marchisio P, Crovari P: **Burden of influenza in healthy children and their households**. *Arch Dis Child* 2004, **89**:1002-1007.
50. Van Der Zalm MM: **Respiratory pathogens in respiratory tract illnesses during the first year of life: a birth cohort study**. *Pediatr Infect Dis J* 2009, **28**:472-476.
